# Zika virus dynamics: Effects of inoculum dose, the innate immune response and viral interference

**DOI:** 10.1371/journal.pcbi.1008564

**Published:** 2021-01-20

**Authors:** Katharine Best, Dan H. Barouch, Jeremie Guedj, Ruy M. Ribeiro, Alan S. Perelson

**Affiliations:** 1 Theoretical Biology and Biophysics, Los Alamos National Laboratory, Los Alamos, New Mexico, United States of America; 2 Center for Virology and Vaccine Research, Beth Israel Deaconess Medical Center, Harvard Medical School, Boston, Massachusetts, United States of America; 3 Ragon Institute of MGH, MIT and Harvard, Cambridge, Massachusetts, United States of America; 4 Université de Paris, IAME, INSERM, Paris, France; 5 Laboratório de Biomatemática, Instituto de Saúde Ambiental, Faculdade de Medicina, Universidade de Lisboa, Lisboa, Portugal; Emory University, UNITED STATES

## Abstract

Experimental Zika virus infection in non-human primates results in acute viral load dynamics that can be well-described by mathematical models. The inoculum dose that would be received in a natural infection setting is likely lower than the experimental infections and how this difference affects the viral dynamics and immune response is unclear. Here we study a dataset of experimental infection of non-human primates with a range of doses of Zika virus. We develop new models of infection incorporating both an innate immune response and viral interference with that response. We find that such a model explains the data better than models with no interaction between virus and the immune response. We also find that larger inoculum doses lead to faster dynamics of infection, but approximately the same total amount of viral production.

## Author summary

The relationship between the infecting dose of a pathogen and the subsequent viral dynamics is unclear in many disease settings, and this relationship has implications for both the timing and the required efficacy of antiviral therapy. Since experimental challenge studies often employ higher doses of virus than would generally be present in natural infection assessment of this relationship is particularly important for translation of findings. In this study we used mathematical modelling of viral load data from a multi-dose study of Zika virus infection in a macaque model to describe the impact of varying the dose of Zika virus on model parameters, and developed a novel mathematical model incorporating viral interference with the innate immune response.

## Introduction

Zika virus (ZIKV), which was the cause of an outbreak in South America that was classified by the World Health Organization as a Global Public Health Emergency in 2016 [1], is a flavivirus primarily transmitted to humans by a mosquito vector although other mechanisms of transmission have been described [2]. Human infection tends to present as a mild fever [3], however associations with neurological [4] and fetal complications [5] have raised concerns about the long-term impact of the outbreak.

A primary experimental readout of the severity of infection with ZIKV and many other viruses is the plasma viral load (VL): the amount of viral RNA present in one ml of plasma. It is thought that the majority of ZIKV infections do not result in clinical symptoms, and when symptoms do occur onset is between 3 and 11 days post exposure [6], hence studying early events in disease progression is difficult in a clinical context. Experimentally, non-human primate (NHP) models of infection recapitulate many features of human infection [7,8] and have been used extensively e.g. [9–13]. In this study we use data from ZIKV infected rhesus macaques [9] to both investigate the role of innate immune response in controlling plasma viremia and assess the effect of inoculum dose on plasma viral load dynamics.

The plasma VL dynamics after infection with ZIKV in the NHP model (e.g. [10]–[13]) mimic those of other acute infections such as influenza [14,15], dengue [16] and West Nile virus [17], with a period of exponential growth until a peak viral load is reached and then a period of exponential decline until the plasma VL becomes undetectable. In a previous modeling study, we did not find evidence for a role of the immune response in controlling the plasma VL since a simpler target cell model fit the available data surprisingly well [18]. In that study the animals were infected with a high dose of ZIKV and we hypothesized that the rapidity of viral spread and target cell destruction via viral cytopathic effects made any restriction of infection due to an immune response difficult to identify in the data.

It is known that ZIKV infection elicits a robust innate immune response, with in vitro studies demonstrating IFN production from a variety of infected cell types [19–21]. Additionally, NHP infection models demonstrate ZIKV-induced changes in innate immune cell concentrations and activation levels [22]. However, the extent of the control that this innate immune response exerts on the acute infection viremia is unclear: ZIKV has been demonstrated to be able to evade the effect of IFN signaling both by degrading STAT2 directly [23] and by interfering with STAT2 and STAT3 phosphorylation [24]. Both STAT2 and STAT3 are intracellular transcription factors that play a key role in the JAK-STAT pathway that leads to the induction of hundreds of interferon-stimulated genes with antiviral effector functions [25].

The choice of inoculum dose in animal infection models is an important aspect of study design. In an experimental setting, higher doses are generally expected to provide more reliable infections, more rapid development of clinically relevant signs and less variability between animals. However, in the case of infections primarily transmitted by a mosquito vector, the natural challenge dose is likely relatively small [13]. How observations in high challenge dose studies can be translated to low natural dose settings is a fundamental question in applying the results of animal model studies to human infection.

The relationship between infection dose and viral dynamics has been explored both experimentally and in mathematical modeling studies. In a mouse model of norovirus infection, higher inoculum doses result in higher and earlier peak viral loads in intestine, mesenteric lymph nodes and spleen [26]. In pigs experimentally infected with foot-and-mouth disease virus, higher inoculum doses give faster viral dynamics and earlier viremia [27]. In a mathematical modeling study of data from a similar foot-and-mouth disease virus experiment in pigs, it was found that differences in viral dynamics after infection with different doses could be accounted simply by altering the parameter describing the initial viral load [28].

A thorough mathematical modeling study of dose effects [29] analyzed viral kinetics in cotton rats after infection with adenovirus type 5 (ADV) [30] and chickens after infection with infectious bronchitis virus (IBV) [31]. Interestingly, the specifics of the dose dependencies were different: for ADV, a higher initial viral growth rate was observed with higher doses while for IBV the inverse was true; and for ADV the peak viral concentration was higher with higher doses while for IBV the peak virus concentration was broadly unaffected by inoculum dose. Testing of different mathematical models found that the observed dose-dependent patterns could not be recapitulated without inclusion of immune responses in the viral dynamics model [29]. A complex relationship between viral dynamics and infection dose is also described in Handel et al. [32], where experimental data and mathematical modeling were used to demonstrate that neither morbidity nor protection from future infection are necessarily monotonic with inoculum dose. A recent study [33] demonstrated that varying inoculum dose provides evidence for innate immune control of viraemia. Combined, these results motivated us to use our multi-dose ZIKV challenge data to see if we are able to detect the signal of an immune response, which could not be identified in our previous single dose study [18]. Further understanding of the effect of inoculum dose on infection dynamics is required for interpretation of results from experimental infection challenges, and here we use data from NHPs after infection with ZIKV to investigate dose-dependent relationships in this experimental setting and to assess whether the role of the immune response in controlling viremia can be determined from these data.

## Results

### Zika plasma viral loads in rhesus macaques

**Fig 1:**
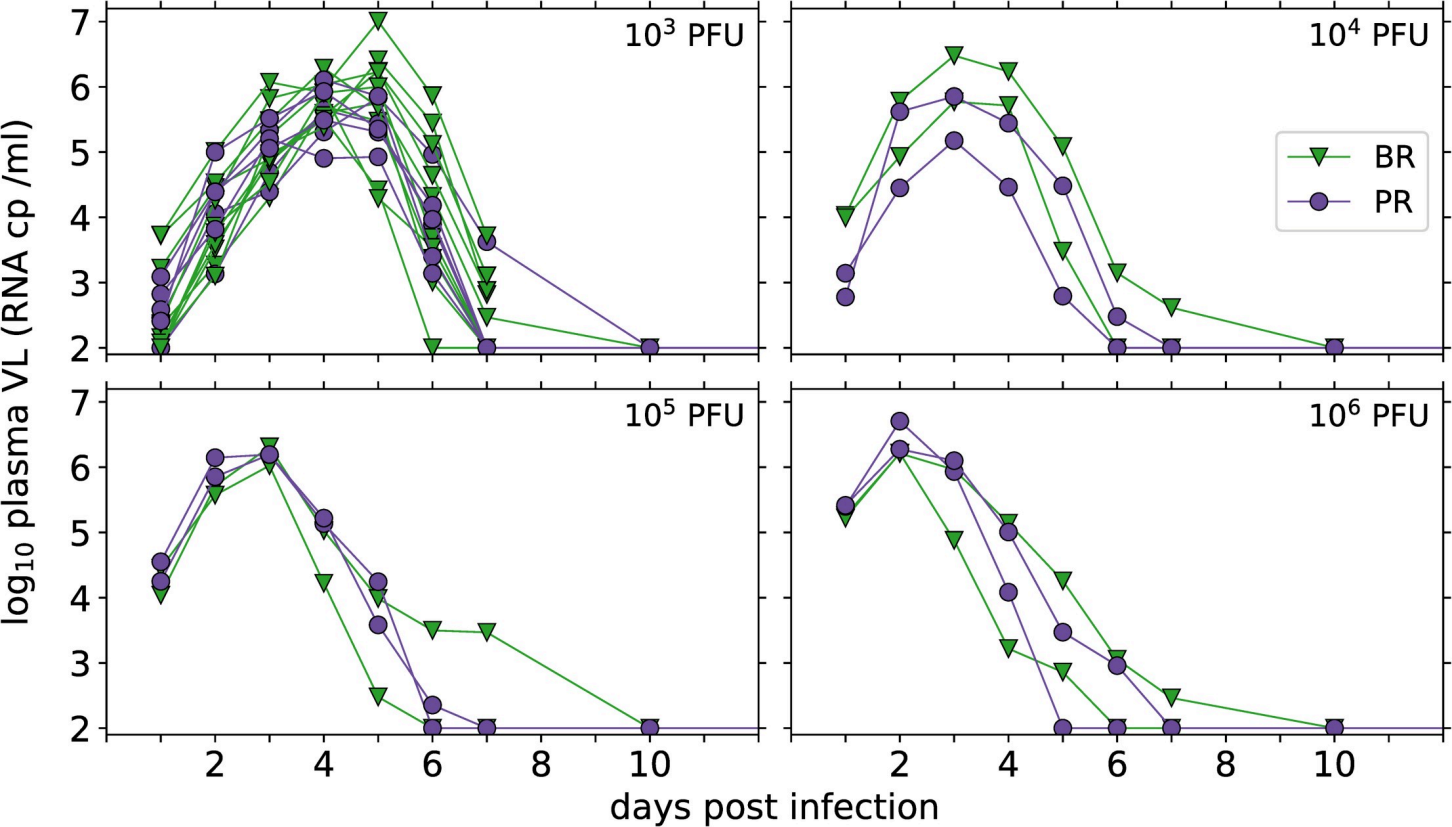
Plasma viral load measurements, in copies (cp) per ml, in rhesus macaques inoculated subcutaneously with the indicated dose of Brazilian (BR, green triangles) or Puerto Rican (PR, purple circles) ZIKV. The limit of detection of the experimental assay is 100 RNA cp /ml, and when ZIKV was undetectable in a sample it is shown here at the limit of detection for illustrative purposes.

**Fig 2:**
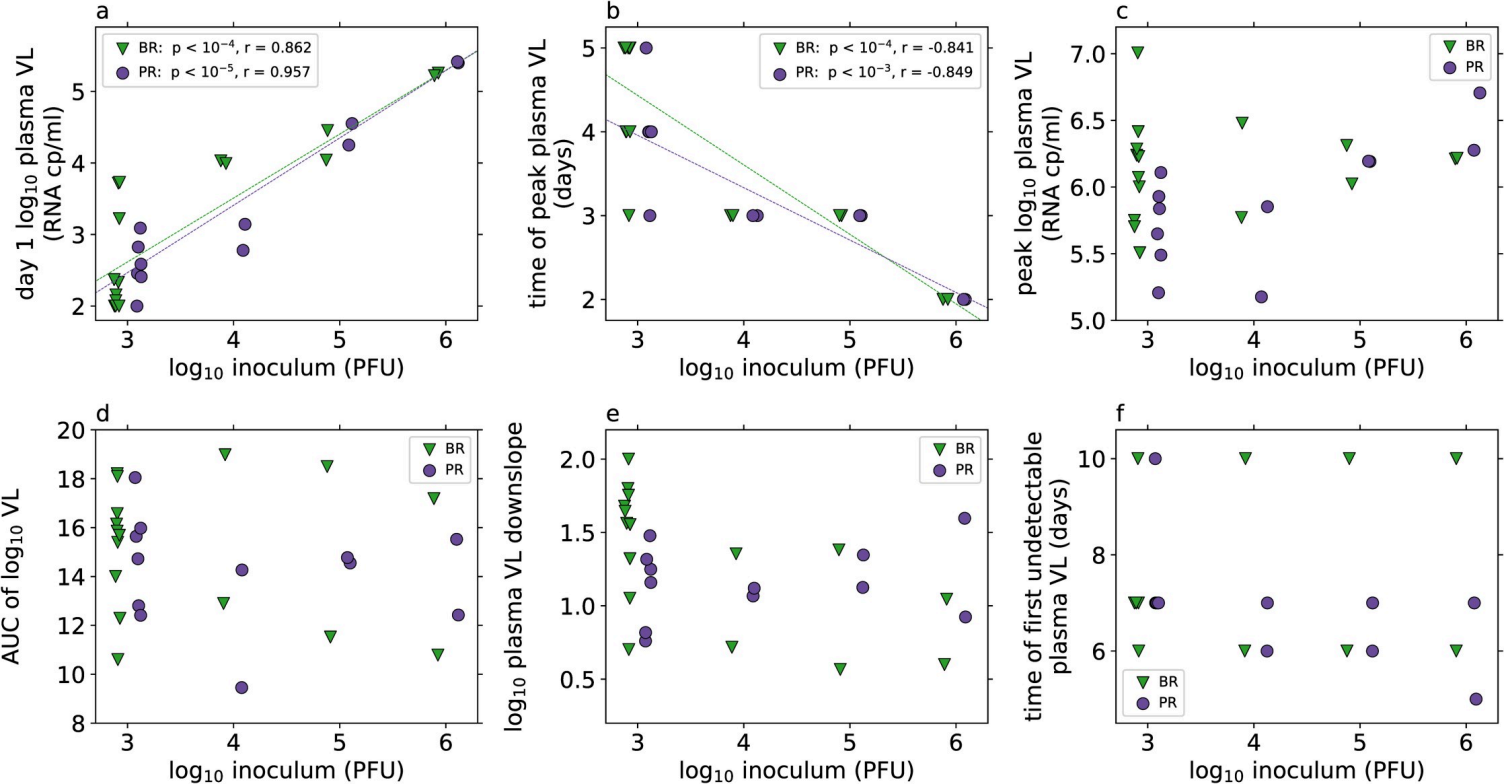
Viral load (VL) characteristics (from measured data, Fig 1) by inoculum dose (log_10_ PFU, x-axis) and viral strain (indicated by markers, BR: green triangles, PR: purple circles). Correlations between inoculum dose and VL characteristics are tested by Pearson correlation for each strain separately, and where this is found to be significant at the α = 0.05 level after Bonferroni correction for multiple testing (*m* = 6) the linear regression line is shown in the panel, and the *p*-value and correlation coefficient are shown in the legend. Differences in VL characteristics between viral strains for each inoculum group are tested with the Mann-Whitney U test, and none are found to be significant at the α = 0.05 level after Bonferroni correction for multiple testing (*m* = 6). The day 1 VL, time of peak VL and value of peak VL are the observed measurements. The area under the curve (AUC) of the log_10_ plasma VL and the downslope of the log_10_ plasma VL are calculated as described in the methods. Animals with VL data only to day 7 or which do not have any undetectable VLs after the peak are excluded from the analysis of “time of first undetectable plasma VL”.

In the data set we analyze, 28 rhesus macaques were infected subcutaneously with 10^3^, 10^4^, 10^5^ or 10^6^ PFU of Brazilian (BR, Brazil-ZIKV2015, Genbank KU497555) or Puerto Rican (PR, PRVABC59, Genbank KU501215) strains of ZIKV, as reported by Aid et al. [9]. Measured plasma viral loads (VLs, Fig 1 and S1 Table) showed that the level of viremia 1 day after infection was dependent on the inoculum dose (Fig 2A), and that higher inoculum dose resulted in a shortened time to peak VL (Fig 2B). There were no other statistically significant relationships observed, either with inoculum dose or viral strain (Fig 2).

The area-under-the-curve (AUC) of the log_10_ plasma VL kinetics can be used as a proxy for the total viral shedding. Interestingly, we found no relationship between AUC and inoculum dose or viral strain (Fig 2D), in contrast to some other infection contexts [29]. This lack of dose-dependence is consistent with, although not conclusive evidence for, a model where viral control is obtained through ‘resource’ limitation, where in this case the resource can be thought of as target cells available to be infected by the virus. In previous work focused on analyzing Zika viral kinetics after high dose inoculation, we showed that a target cell limited model described infection dynamics well [18], consistent with no observed relationship between AUC and inoculum dose. Here we extend these analyses to a larger dataset, focusing on whether studying multiple inoculum dose simultaneously provides any evidence for immune effects.

### Target cell limited model

Mathematical models based on target cell limitation have been used to describe acute infection plasma viral loads in HIV [34,35], influenza A [14], West Nile virus [17] and Zika virus [18]. In the target-cell limited model described by the ordinary differential equations (ODEs) in Eq 1, target cells, *T*, are infected by free virus, *V*, under a mass-action like process with infection rate constant β. The infected cell population in this model is split into two classes of cells: *I*_1_ cells that do not produce virus and are referred to as being in the eclipse phase, which transition at per capita rate *k* into *I*_2_ cells that are productively infected. The productively infected cells die at per capita rate δ. It is assumed that since *I*_1_ cells do not produce virus they are not subject to viral cytopathic effects and no death of this cell class is modelled. Productively infected cells produce free virus, at rate *p*, which is degraded at per capita rate *c*. We assume that, over the timescale of the acute infections considered here, production and loss of target cells by non-infectious processes roughly balance and hence are neglected in the following model:

$$\begin{array}{l} dT/dt=-\beta VT,\;T(0)=T_{0}\\ dI_{1}/dt=\beta VT-kI_{1},\;I_{1}(0)=0\\ dI_{2}/dt=kI_{1}-\delta I_{2},\;I_{2}(0)=0\\ dV/dt=pI_{2}-cV,\;V(0)=V_{0} \end{array}$$ (Eq 1)

Target cell limited models, either with or without the eclipse phase as modelled here, have the property that the VL AUC is determined primarily by the number of target cells available, independently of the initial dose [15]. Modeling an eclipse phase, which allows for a biologically necessary amount of time between infection and viral production, introduces an additional parameter to describe the rate of transition from the eclipse phase to productive infection and as such this might not be supported by model selection theory. However, including an eclipse phase makes the model more biologically realistic and in a model of influenza, it was found that incorporating an eclipse phase provided a more reasonable estimate of the life span of infected cells [14].

We used non-linear mixed effects modeling, as described in Methods, to fit the plasma VLs in all 28 monkeys simultaneously and obtain estimates of the population distributions for model parameters. We found that models incorporating an average eclipse phase length of four hours or shorter (*k* ≥ 6 d^-1^) and a viral clearance rate *c* ≥ 5 d^-1^ generally gave indistinguishable model fits (S1 Fig). Although the overall fitting criterion was very stable with these choices of *k* and *c*, the estimated population parameters vary somewhat (S2 Fig). To investigate the presence of dose-dependent relationships and the effect of immune responses in these data, we chose to use fixed *k* = 8 d^-1^ and *c* = 10 d^-1^ and based further analysis on the best fit we found with these parameter values. We note, however, that there is no specific support for these values of *k* and *c* over other fixed values shown in S2 Fig.

Despite not explicitly including any effect of inoculum dose in this model fit, we found that the estimated initial plasma viral load, *V*_0_, for individual animals is tightly correlated with the inoculum dose administered (S3 Fig, *p* < 10^−10^). The viral inoculum in these experiments was administered subcutaneously, while the VL measurements were taken in the plasma. As such the estimated *V*_0_ represents the effective initial inoculation size in the plasma, after transport from the inoculation site rather than being directly comparable to the inoculum dose. In this model, no other statistically significant relationships were found between individual parameter estimates and inoculum dose (S3 Fig) and no differences in parameter estimates were observed between the two viral strains (S4 Fig).

As a more thorough analysis of the effect of inoculum dose or viral strain on the viral dynamics parameters, we allowed population parameters to depend on dose or strain as covariates in the model fitting. We then used an iterative approach to determine which covariate relationships should be retained, as described in Methods. This approach accepts covariate structures that describe statistically significant relationships between dose and parameter value while maintaining at least as good a model fit. We found statistical support only for a relationship between estimated initial plasma viral load, *V*_0_, and inoculum dose (S2 Table). Including this covariate relationship in the fitted model (S5–S9 Figs) gave statistically significantly improved log likelihood of -169.2 (compared to -194.3 without any covariates, p value < 10^−10^ by the log likelihood ratio test) and reduced the estimated standard deviation of log_10_ V_0_ from 1.18 to 0.33 (Table 1).

**Table 1:** Estimated population parameters from fitting the indicated models to viral load data from all animals using a non-linear mixed effects model. An explicit covariate relationship between inoculum dose and initial viral load *V*_0_ is incorporated, with the median population estimate for log_10_ *V*_0_ at each inoculum dose shown in italics. Relative standard errors are shown in parentheses. Fixed parameters used in the model fits are: *k* = 8 d^-1^, *c* = 10 d^-1^, *s* = 1 d^-1^, α = 2 d^-1^, *K* = 10^3^, *n* = 0.25, *T*(0) = 10^5^ ml^-1^, each with no variability.

	Target cell limited model (Eq 1)				Innate immune model (Eq 2)				Viral interference model (Eqs 2 & 3)			
Parameter	Population estimate		Variability estimate		Population estimate		Variability estimate		Population estimate		Variability estimate	
*R*_*0*_	2.59	(16%)	0.0229	(572%)	3.44	(12%)	0.0284	(888%)	4.16	(9%)	0.033	(405%)
δ	8.83 d^-1^	(57%)	0.211	(60%)	3.31 d^-1^	(21%)	0.116	(103%)	2.25 d^-1^	(10%)	0.064	(85%)
*p*	1724 d^-1^	(65%)	0.596	(33%)	637 d^-1^	(24%)	0.536	(24%)	450 d^-1^	(16%)	0.514	(26%)
γ					3.36	(49%)	0.859	(52%)	0.010	(59%)	2.12	(24%)
τ					5.37 d	(5%)	0.239	(15%)	2.68 d	(3%)	0.107	(29%)
log_10_ *V*_*0*_	-1.51 ml^-1^	(23%)	0.329	(28%)	-1.00 ml^-1^	(8%)	0.341	(26%)	-0.96 ml^-1^	(34%)	0.359	(21%)
*at 10*^*3*^ *PFU*	*1*.*50 ml*^*-1*^				*1*.*91 ml*^*-1*^				*1*.*95 ml*^*-1*^			
*at 10*^*4*^ *PFU*	*2*.*50 ml*^*-1*^				*2*.*87 ml*^*-1*^				*2*.*92 ml*^*-1*^			
*at 10*^*5*^ *PFU*	*3*.*50 ml*^*-1*^				*3*.*84 ml*^*-1*^				*3*.*89 ml*^*-1*^			
*at 10*^*6*^ *PFU*	*4*.*51 ml*^*-1*^				*4*.*81 ml*^*-1*^				*4*.*86 ml*^*-1*^			

We noted that the parameter estimates for the death rate of productively infected cells, δ, gave a population median of 8.8 d^-1^ (S6 Fig), providing an average estimated lifespan of a productively infected cell of less than 3 hours, and a range in individual estimates between approximately 6 d^-1^ and 11 d^-1^. These estimates were much higher under this model fit than has been found for other viruses [14,36,37]. Although there is a substantial amount of uncertainty in the estimate of this parameter (Table 1), we found that restricting the maximum death rate of productively infected cells to < 6 d^-1^, in line with estimates for other viruses, generally reduced the quality of the model fit (S10 Fig).

### Modelling immune control of plasma viremia

The target cell limited model (Eq 1) is able to provide a good description of the observed viral load data (S5 and S9 Figs) but does not provide relevant insight into the immune response and mechanisms of control of plasma viremia. Under the target cell limited model, infection is controlled only when there is substantial depletion of target cells. Given the breadth of cell types that ZIKV is able to infect [19,21,38–42] it is likely that a model relying on substantial depletion of all target cells will be missing important biological mechanisms. Innate immune responses can rapidly restrict viral infections and in these macaques interferon-stimulated genes were upregulated following infection [9]. In previous work, using an inoculum dose of 10^6^ PFU, we found no evidence for immune control of Zika plasma viremia [18] when incorporating measured immune cell or cytokine dynamics, including type I interferon (IFN), into the model. In that study, with a high viral challenge the resultant viral kinetics were rapid. In the data analyzed here the different inoculum doses result in a range of times to peak VL after infection (Figs 1 and 2) possibly providing more information on early viral replication and the mechanisms of innate immune control of plasma viremia.

We extended the viral dynamic model given by Eq 1 to incorporate an explicit innate immune effect on plasma viremia and tested whether this additional complexity could be supported by the data. We modelled the effect of an innate immune response, *X*, which could be thought of as a cytokine such as IFNα, or a combination of cytokines, that act to alter the virus dynamics. As in the work of Baccam et al. [14] on the innate response to influenza infection, we assumed this generic innate immune response factor is produced at a rate proportional to the concentration of productively infected cells, after a time delay, and decays at a constant rate (Eq. S1). However, we note that the dynamics of *X* are not necessarily comparable to the dynamics of soluble IFNα or other cytokines, and instead *X* should be considered as a measure of the effective cytokine concentration rather than the measurable cytokine concentration since ZIKV can interfere with cytokine signaling, including through the JAK/STAT pathway [23,24,43,44].

There are a number of ways that the action of the innate immune response can be included in the mathematical model. We initially tested models where the innate response affects the viral dynamics in one of a number of ways: (i) reducing viral infectivity (Eq. S2), which also models making target cells less susceptible to infection, (ii) increasing the death rate of infected cells (Eq. S3), a possible action of natural killer cells or (iii) reducing the rate of viral production from infected cells (Eq. S4), an activity of IFNα when used to treat hepatitis C virus infection [45,46]. Under our initial testing criteria each of these models provided a statistically better fit to the observed data than the target cell limited model (S1 Text and S3 Table). Allowing the immune response to reduce the rate of viral production from infected cells provided the best fit in this initial test and therefore we further analyzed this model.

The model where the innate immune response reduces the rate of viral production from infected cells is described by the following system of ODEs:

$$\begin{array}{l} dT/dt=-\beta VT\;,\;T(0)=T_{0}\\ dI_{1}/dt=\beta VT-kI_{1},\;I_{1}(0)=0\\ dI_{2}/dt=kI_{1}-\delta I_{2},\;I_{2}(0)=0\\ dV/dt=\hat{p}I_{2}-cV,\;V(0)=V_{0}\\ dX/dt=sI_{2}(t-\tau )-\alpha X,\;X(0)=0\\ \hat{p}=p/(1+\gamma X) \end{array}$$ (Eq 2)

Without loss of generality and for the sake of identifiability we set the coefficient of production of the innate response factor, *s*, equal to 1 d^-1^ such that *X* is in units of daily production. This innate immune response model then contains three additional parameters to be estimated: the delay in production of the immune response factor (τ), the rate of degradation of the immune response factor (α), and the strength of the immune response factor in modulating viral production (γ). The available VL data do not provide enough information to allow all of these parameters to be estimated fully in the population. Instead, the degradation rate α was fixed to be 2 d^-1^, approximately equal to the value found in a modelling study of influenza in ponies [47], and population distributions for τ and γ were estimated.

Fitting the innate immune model (Eq 2) to the plasma VL data (S11–S15 Figs, Table 1 and S4 Table), including estimating distributions for γ and τ, gave statistical support for this model over the target cell limited model via the log likelihood ratio test, with a maximum log likelihood of -148.5 (compared to -169.2 for the best target cell limited model, *p* < 10^−6^ by the log likelihood ratio test with six degrees of freedom), providing evidence for a process other than target cell limitation as a mechanism helping control of plasma viremia in these data. We also note that the innate immune model provides different estimated parameter distributions than the target cell limited model (S5 Fig compared to S12 Fig). In particular, the innate immune model provides lower estimates of the infected cell death rate, δ, more in line with those of other viral infections [14,36,37]. We found that the estimated parameters are stable to the choice of the fixed value of α, the rate of degradation of the immune response (S16 Fig).

When considering the individual estimated parameters under this model, we didn’t observe any statistically significant relationships between parameter values and inoculum dose or viral strain (S13 and S14 Figs), other than the explicitly incorporated relationship between estimated initial viral load and dose. In the same manner as for the target cell limited model, we again thoroughly assessed possible inoculum dependencies in parameters by testing each possible covariate relationship in the model fitting procedure (S5 Table). Inclusion of an explicit relationship between the timing of the initiation of the immune response, τ, and the inoculum dose is supported under our selection criteria (see Methods), with a p-value from the Wald test of < 10^−3^, significant after Bonferroni correction for multiple testing, and a log likelihood of -146.17 (compared to -148.5 for the model without this covariate relationship, giving a p-value of 0.03 under the log likelihood ratio test with 1 degree of freedom). These results suggest the possibility of some additional dose dependent mechanisms that are not currently being accounted for in our mathematical model.

Fitting the model including a dose dependency in τ (S17–S21 Figs and S6 Table) resulted in an estimated covariate coefficient B_τ_ = 0.178 (see Methods). Perhaps surprisingly this indicates a positive relationship between inoculum dose and time of initiation of the immune response, τ, such that this innate immune response model predicts that higher doses result in a longer delay in immune response. Despite some statistical support for the inclusion of a dose dependency in the timing of the immune response there is not an obvious biological mechanism to explain this relationship. We speculated that this observed dose dependency was in fact due to limitations of the current mathematical model and hypothesized that the result could be indicative of the ability of ZIKV to subvert the IFN response through the STAT signaling pathway [23,24]: at higher inoculum doses this interference might be more successful and in the current model structure this is being accounted for as a longer delay before the immune response takes effect. The biological observation of viral interference with immune response motivated the development of an extended mathematical model to describe how this mechanism might act on the viral dynamics.

### Viral interference of host immune response

To mechanistically describe the ability of ZIKV to subvert the innate immune response in the context of our viral dynamics model we modified the form of the immune restriction of viral production, rewriting $\hat{p}$ in Eq 2 as:

$$\hat{p}=p/(1+\gamma X(1-g(V)))$$

with *g*(*V*) = 0 corresponding to the model in Eq 2. The function *g*(*V*) takes values between 0 and 1, with *g*(*V*) = 0 indicating no viral interference in the immune response and *g*(*V*) = 1 indicating complete viral suppression of the innate response effect on viral production. We describe viral interference in the immune response with a Hill function type expression:

$$g(V)=V^{n}/(K^{n}+V^{n})$$ (Eq 3)

such that at low viral loads *g*(*V*) is close to 0 (no viral interference) and when the viral load *V* is much larger than the parameter *K* we find that *g*(*V*) becomes close to 1 and the viral production rate $\hat{p}$ is less affected by the immune response *X* (S22 Fig). The parameter *K* determines the level of viral load required for 50% viral interference and the parameter *n* determines how sharply the level of interference increases with viral load (S22 Fig).

This form of the reduced viral production rate $\hat{p}$ introduces two additional parameters which are unable to be well estimated from the available viral load data. Instead, we tested the model fit with different fixed values of *K* and *n*, allowing the initial viral load, *V*(0), to depend on the inoculum dose but requiring that timing of the immune response, τ, follows the same distribution for all animals. Taking *n* = 0.25 or 0.5 and *K* between 10^0^ and 10^3^ provides indistinguishable fits by log likelihood (S23 Fig). For further analysis we selected the model fit with *K* = 10^3^ and *n* = 0.25 (Table 1 and S24–S28 Figs), which has a log likelihood of -145.0 and provides a slightly better description of the observed viral loads than the model with *g*(*V*) = 0 (log likelihood ratio test with 2 degrees of freedom to account for *K* and *n*, p = 0.03).

We again tested each possible additional covariate relationship in this model and under our selection criteria (see Methods) found no statistically significant relationships between inoculum dose or strain and model parameter values (S7 Table), except for the included dependency of *V*_0_ on inoculum dose.

**Fig 3:**
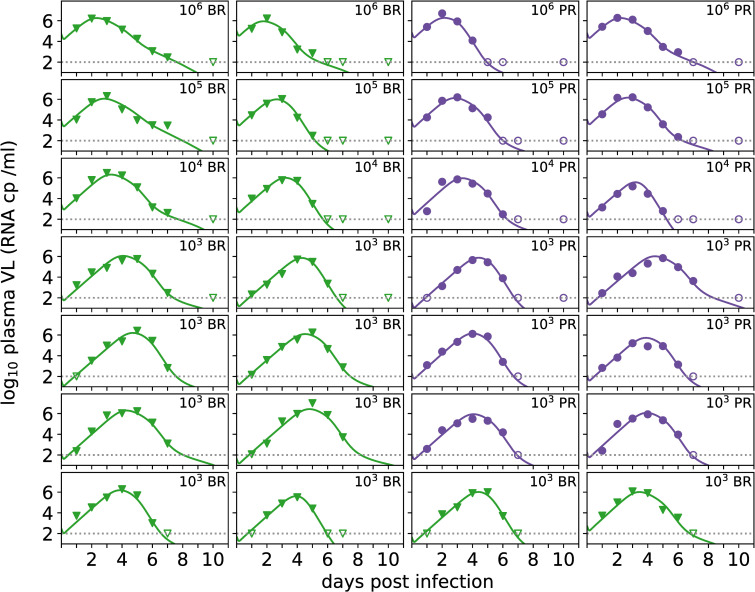
Individual viral load predictions from population model fits. For each animal the observed plasma viral loads (markers), the predicted viral load from the immune response model with viral interference (Eq 3, Table 1) are shown. The horizontal grey dotted line represents the limit of detection of the assay, and samples in which virus is not detected are shown with open markers at this value. Viral strain is indicated by marker and color (BR: green triangles, PR: purple circles) and both inoculum dose and viral strain are indicated in the top right corner of each panel.

This viral interference model describes the individual viral dynamics in each animal well (Fig 3) and provides a statistically significant improvement in fit after accounting for additional parameters over the innate immune model without viral interference, as well as over the target cell limited model which relies solely on resource depletion to account for viral control. Adding the known biological phenomenon of viral interference with immune response allows for more insight into the effect of both host response and viral evasion of that response, as well as providing a somewhat improved fit of the model to the data.

**Fig 4:**
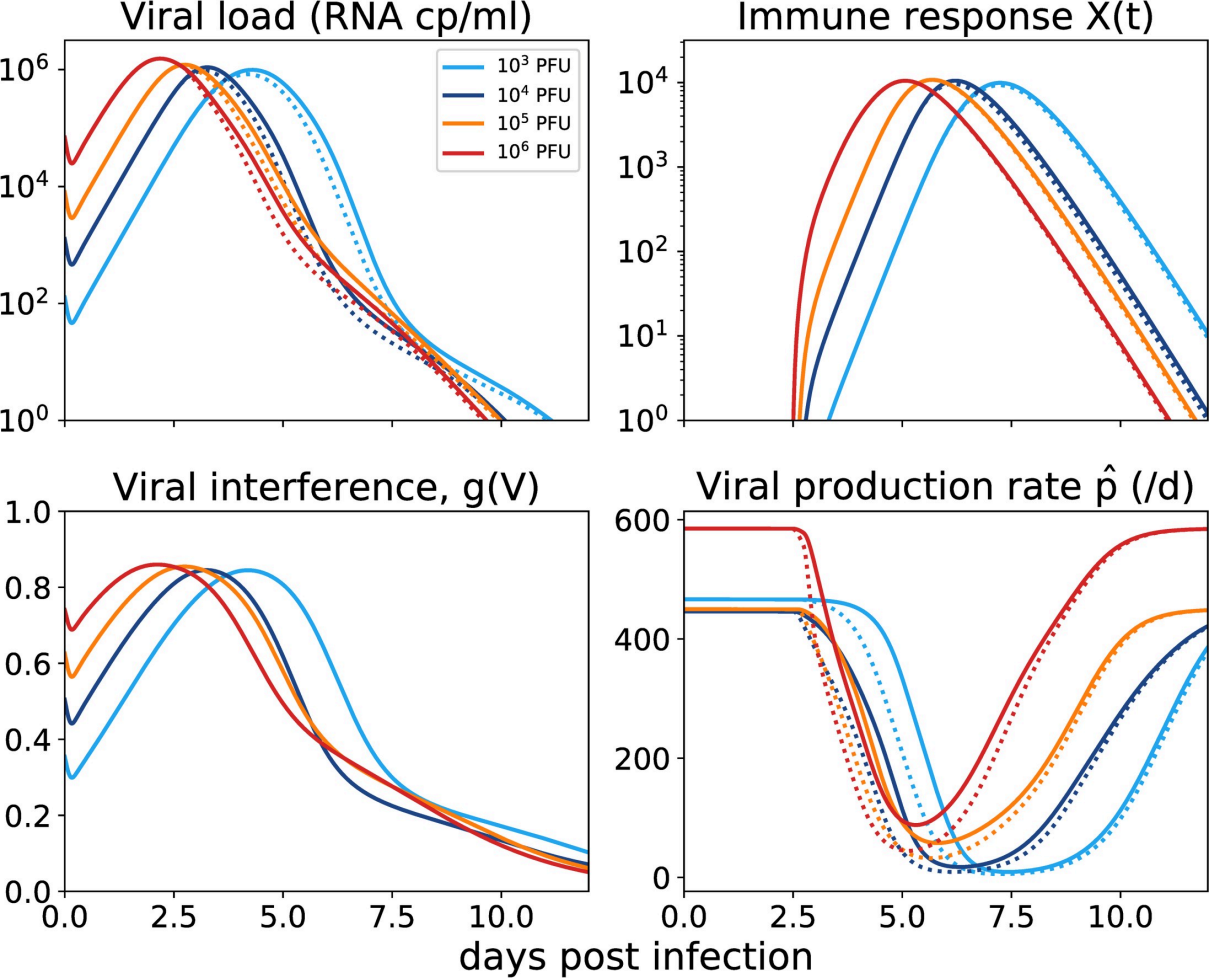
Predicted dynamics for the immune response model with viral interference (Eqs 2 and 3, Table 1). Each inoculum dose is indicated by color, with the mean predicted dynamics within an inoculum group shown. The solid line shows the model dynamics with the estimated parameters. The dotted line shows the model dynamics with estimated parameters but with *g*(V) fixed at 0, such that viral interference has no effect on immune response.

In Fig 4, we show the effects of the predicted immune response on virus and viral production. We can now clearly see that higher inoculum results in faster stimulation of the immune response (*X*), but also a stronger viral interference effect *g*(*V*) early on, which overall results in larger daily viral production. This indicates that, as well as improving the model fit to the data, this viral interference mechanism removes the biologically strange delayed immune response phenomenon observed with the simpler immune response model.

The fraction $\hat{p}/p$ can be used as a measure of the extent to which the innate response is able to reduce the viral production rate, with a value of 0 meaning complete suppression of viral production and a value of 1 meaning no effect of the immune response. At higher inoculum doses the area under the curve of $\hat{p}/p$ is higher (S29A Fig), indicating that higher doses result in a less efficient innate immune response overall. Similarly, immune-mediated viral restriction ($1-(\hat{p}/p),$ S29B Fig) is lower when the inoculum dose is higher. However, despite the ability of higher inoculum doses to subvert the immune response, the total viral production is not related to dose (S14C Fig) as suggested by the observed lack of correlation between inoculum dose and viral load AUC (Fig 2). The fact that total viral production is not related to dose may simply reflect that at higher doses with the innate immune response partially subverted viral load peaks early and then rapidly declines to undetectable levels as cells susceptible to infection are depleted (S30 Fig).

**Fig 5:**
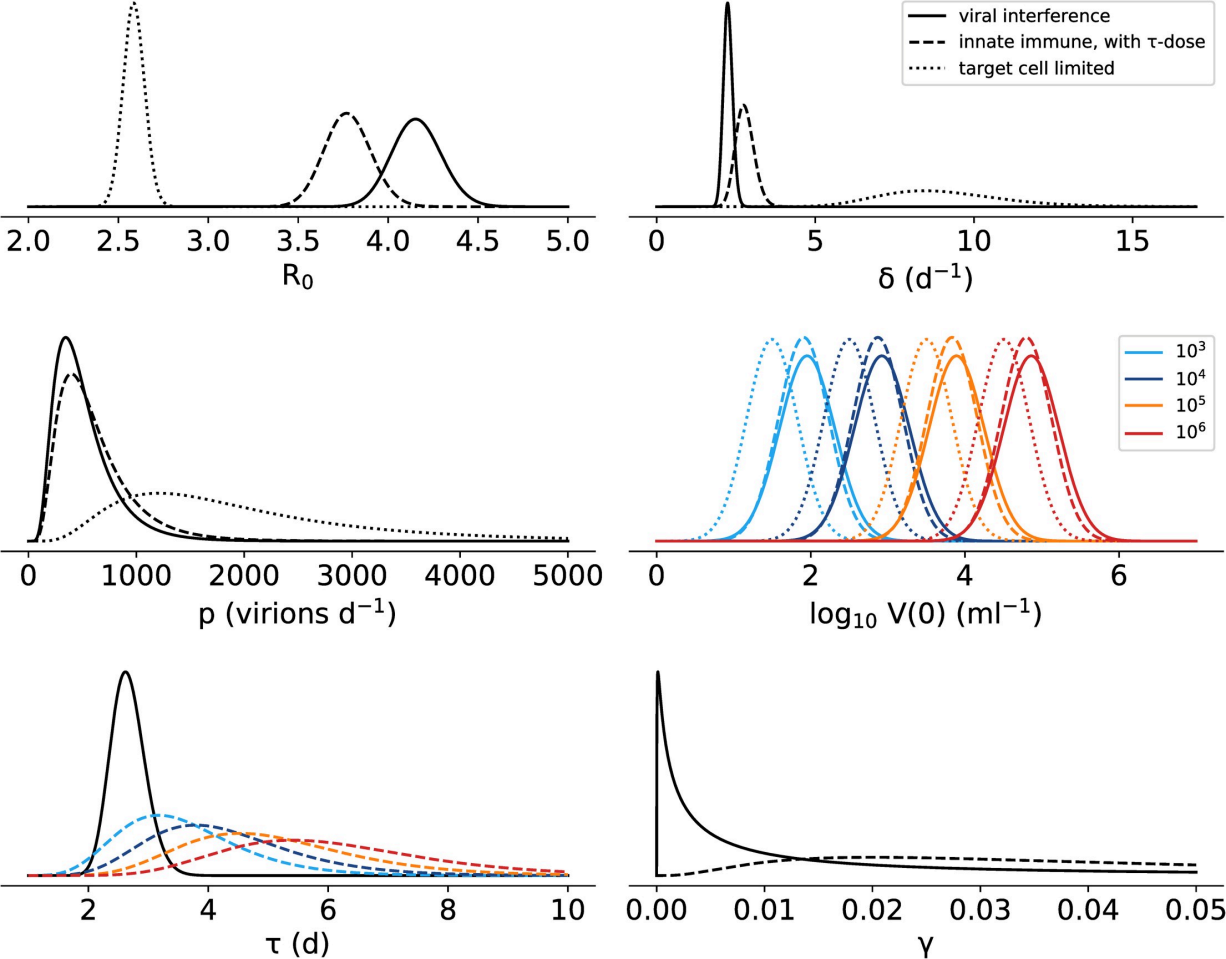
Estimated parameter distributions for the three model fits: immune response model with viral interference (Eq 3, Table 1, solid line), the innate immune response model including a dose dependency in the timing of the immune response (Eq 2, S6 Table, dashed line) and the target cell limited model (Eq 1, Table 1, dotted line). Where inoculum dose is explicitly included as a covariate in the model, the distributions for each dose are indicated by color.

The estimated parameter distributions from each of the models considered in this study (Fig 5) show that the estimated infected cell death rate is much lower in the innate immune models than in the target cell limited model. The estimate for the time delay in the innate immune response is reduced to a median 2.7 d when viral interference is included in the model, but this estimate is still longer than the time when we were able to detect soluble interferons in circulation in our previous ZIKV study [18]. However, we interpret this as suggesting that the effect of the immune response is delayed from the production of the cytokines, as ZIKV does not affect the production of cytokines but rather their signaling through the JAK/STAT pathway. Alternatively, it could be that the timing of the immune response is also subject to viral interference which is unaccounted for in our modelling. Fitting the model with no time delay in the immune response reduces the quality of the fit: across a range of values tested for log_10_ K and n, the maximum log likelihood we achieved with τ = 0 was -154.4, compared to a log likelihood of -145.0 with the best-fitted τ.

The only dose dependency that is supported in the viral interference model is in the initial viral load *V*(0), and the observed relationships between dose and day 1 plasma viral load and time of peak viral load (Fig 2) are captured by the model without requiring any additional dose-parameter relationships (S31 Fig). Interestingly, in the simulated data we see a statistically significant relationship between inoculum dose and the area under the viral load curve as well as between inoculum dose and the time viral load first becomes undetectable, suggesting that these relationships may be present in the observed data, but without enough power to be statistically significant. These additional relationships suggest that higher inoculum doses result in plasma viral load dynamics with a faster time to undetectable and a (very slightly) lower total viral burden. However, given the extremely shallow relationship between dose and area under the curve it’s unlikely that this relationship would have any translational effect in animal models.

## Discussion

Experimental challenge models are used to study many viral infections, and in order to obtain robust infections it is often the case that the inoculum doses used are many times higher than exposure in a natural setting. In order to translate findings from experimental to natural infection settings the impact of the inoculum dose needs to be understood.

Here we studied the effect of inoculum dose on plasma viral loads (VLs) after Zika infection of non-human primates. We found dose-dependent behavior in the viral load dynamics that is not readily apparent from summary statistics of the VL measurements, demonstrating the value of careful mathematical modelling analyses.

The subcutaneous viral inoculum dose given (measured in PFU) was found to be well correlated with the estimated effective initial viral load in circulation (measured in RNA cp/ mL), and across the dose range used in this study we saw no evidence of any saturation in the transport process from tissue to circulation. With an appropriate transformation, the log_10_ inoculum dose can be considered to be equivalent to the initial log_10_ viral load in the model (e.g S3 and S5 Figs).

The data presented in this analysis, in contrast to our previous study [18], statistically supports a mathematical model of an IFNα-like immune response to viral infection, reducing the rate at which infected cells are able to produce new viral particles. However, the experimental data doesn’t include any kinetic measures of the immune response at early timepoints after infection, restricting our analysis to a heuristic model of the innate immune response. The VL data alone is not sufficient to identify the dominant mechanism of immune response: here we primarily worked with a model where the immune response restricts viral production, but other models where the immune response increases the rate of clearance of infected cells, reduces the infectivity of the virus, or protects target cells from infection are all also statistically supported above the target cell limited model, although do not give as good a fit to the observed data as the model with reduction in viral production. In order to more clearly understand control of Zika virus in mild acute infections further careful data collection will be required. In particular, the relationship between soluble cytokine concentration and the magnitude of the effect of the cytokine is not necessarily linear.

The statistical approach of estimating population parameter distributions that was used in this study allows for an analysis of whether the inoculum dose size has an influence on any of the model’s parameter values. We saw that introducing a relationship between the inoculum dose and the time delay in the immune response improved the model fit and was statistically significant. The time delay in the model can be thought of as the time it takes for the appropriate cytokine signaling, cytokine production and the cytokine’s effect to occur after a cell becomes infected. The predicted relationship between this time delay and the inoculum dose was such that at higher doses there was a longer delay. Rather than reflecting a true biological dose-dependence, we took this relationship as providing clues for additional mechanisms to include in the model. Zika virus is able to interfere with the host immune system, and modeling this interference with a Hill function-like dependency on viral concentration allowed us to describe the observed data better.

Our novel viral interference model is able to capture more of the biology of the dynamics seen in these acute infections. It removes the surprising relationship between inoculum dose and the timing of the immune response seen with the innate immune response model, as well as providing a framework for a quantitative description of the effect of the experimentally observed degradation of cytokine signaling by ZIKV.

However, we were not able to accurately identify the shape of the relationship between viral load and suppression of the immune response. The half-maximal concentration was seen to be able to range over 3 orders of magnitude while providing equivalently good fits, and Hill coefficients of 0.25 and 0.5, meaning shallow slopes in the Hill function *g*(*V*) (S22 Fig), both gave good descriptions of the observed viral loads (S23 Fig). Hill coefficients of less than 1 are usually interpreted, in a biochemical reaction setting, as negative co-operation. How this parameter value should be interpreted in the setting of viral interference is less clear. A Hill coefficient of less than 1 gives behavior in the viral interference expression *g*(V) where the per-virion interference, *g*(V)/V, decreases as viral concentration increases even though *g*(V) is monotonically increasing. At lower viral concentrations, early in infection or after low inoculum doses as in a natural infection, the per-virion interference with immune response is more effective than when the viral load is higher.

In our mathematical model, the viral concentration exerts effects on itself, beyond the simple viral replication through cell infection, via two competing mechanisms. First, free virus infects target cells in a concentration dependent manner and the infected cells initiate an immune response that restricts production of new free virus. Second, free virus is able to inhibit the immune response through the viral interference mechanism, increasing viral production back towards the baseline rate. The combined impact of these two mechanisms on the course of infection will be complex and depends on both the timing and magnitude of the immune response and viral interference.

These two mechanisms do not depend on viral concentration in the same manner. As discussed above, the per-virion effect of interference decreases with higher viral concentrations, while the per-virion effect of immune response does not vary with viral concentration although there is a complicating factor of the time delays in the immune response. These differing concentration dependencies mean that the ratio of immune response effect to viral interference is more heavily weighted towards the viral interference mechanism at low viral loads than at high viral loads. In natural infection settings, the initial viral concentration from a mosquito bite is likely to be substantially lower than in experimental infection models. Dudley et al [13] infected macaques with ZIKV via mosquito bite, with mosquitos that were allowed to feed on infected mice 12 days previously. They found that immediately after infecting the macaques, the mosquito saliva contained approximately 10^2^ PFU of ZIKV, compared to a lowest inoculum dose of 10^3^ PFU in the experimental infection data examined in the current study. Since viral loads in infected macaques are likely to be lower early in natural infection compared to high dose experimental infections, it is feasible that viral interference of the innate immune response is a key mechanism in natural infection.

This framework for considering the effect of the immune response on viral dynamics might have an impact on assessments of antiviral efficacy from high dose experimental challenge models. A treatment that reduces viral load will also presumably reduce both the innate response and viral interference with the innate response. The variable balance of the effect of these mechanisms at a lower challenge dose might change the effectiveness of the therapy. The dynamics of the host immune response, and how the virus interferes with it are important to uncover in order to further our understanding of how ZIKV infection is usually effectively controlled.

## Methods

### Data

Plasma viral load (VL) measurements, assessed using an RT-PCR assay, were collected after subcutaneous infection with ZIKV as described in [9]. They were analyzed as log_10_ VL, and for visualization purposes when ZIKV was undetectable in a sample, the data point is displayed at the experimental limit of detection, 100 RNA copies /mL. Area under the curve (AUC) was calculated via a trapezoid method on the log_10_ viral loads above the detection limit (log_10_ viral load minus log_10_ detection limit). The downslope was measured by linear regression on the log_10_ data points between the peak viral load and the first undetectable sample, inclusive. Where there was no undetectable sample after the peak viral load, in the few animals where samples were only taken to day 7, the data points between the peak viral load and the last measured viral load were used for linear regression.

### Statistical significance

Statistical significance was assessed with a threshold of α = 0.05, accounting for multiple testing via the Bonferroni correction where appropriate.

### Mathematical models

Ordinary differential equation (ODE) based compartmental models were used to describe the plasma viral dynamics, as detailed in the text.

#### Model fitting approach

As in our previous Zika modeling work [18], we used a non-linear mixed effects modeling approach, implemented in Monolix 2016 (lixoft.com/products/monolix/) in which the VL data from all animals, at all doses, was fitted simultaneously, on a log_10_ scale. In this modeling approach each estimated parameter is assumed to follow a log-normal distribution in the population, and the parameter value for an individual *i* can be expressed as $\theta_{i}=\theta e^{\eta_{i}}$ where θ is the median value of the population distribution and η_i_ is the individual random effect, assumed to be normally distributed as *N*(0, ω^2^), accounting for variability between individuals. We note that we did not fit or enforce in the statistical model structure any correlations among parameters. For model fitting in Monolix we use settings: SAEM k1 = 1000, k2 = 200, MCMC chains = 4, estimation of the Fisher information matrix (FIM) by linearization and log-likelihood (using natural logarithms) by importance sampling. For other settings, the defaults were retained. Data below the limit of detection of the experimental assay was handled as censored data [48].

#### Parameters

We typically have seven positive data points per animal, thus it is likely that not all parameters are practically identifiable [49], especially for parameters describing processes that occur on the scale of hours or minutes. For this reason, we chose to test a set of fixed values for the rate of viral clearance, *c*, and for the transition rate from the eclipse phase to productive infection, *k*, instead of trying to estimate these rates by fitting.

For dengue virus, a similarly structured flavivirus, a mathematical modeling study estimated the clearance rate to be around 5 d^-1^ [50]. For West Nile virus, also a flavivirus, measurements of viral loss from serum over 90 minutes after intravenous infection showed a total clearance rate greater than 40 d^-1^, although this rate includes infection of target cells and is therefore an upper bound. For HCV, another flavivirus, the viral clearance rate has been estimated to be around 22 d^-1^ [51]. With the daily sampling schedule in the data used in this study, the viral clearance rate is likely too fast to be estimated well. As such, we tested a range of values of clearance rates between 5 d^-1^ and 20 d^-1^.

Similarly, the length of the eclipse phase is unlikely to be identifiable from these viral load data, since the samples are taken at most daily and the average eclipse phase length is likely to be less than 1 day given the high VLs observed on day 1 in many monkeys. As such, VL measurements within the first day post infection would be required to fully estimate the eclipse phase length. Hamel et al. [19] found detectable ZIKV production in vitro 6 hours after infection of primary human fibroblasts, and analysis of the data from more detailed in vitro studies such as [52] would provide further insight into the eclipse phase length in ZIKV infection. In this study, we tested model fits with fixed eclipse phase transition parameter values between 1 d^-1^ (giving an average eclipse phase length of 24 hours) and 24 d^-1^ (giving an average eclipse phase length of 1 hour).

To select values of *c* and *k*, the fitting algorithm was run for each pair of fixed values at each of 10 randomly generated seeds and 10 randomly generated sets of initial guesses, giving a total of 100 algorithm runs for each tested set of *k* and *c*. Model fits under each pair of fixed values of *c* and *k* were assessed by log likelihood.

Additionally, it is known that in this model only the product *pT*_*0*_ is identifiable from viral load data [34], so we fixed the initial concentration of target cells, *T*_*0*_ = 10^5^ cells ml^-1^, a value used previously [18], and estimated a production rate *p*.

Following Snoeck et al. [53], we chose to reparametrize the model to allow estimation of the basic reproductive ratio, *R*_0_, which represents the number of cells that would be infected by the virus produced from a single infected cell introduced into a fully susceptible target cell population. *R*_0_ in the target cell limited model with eclipse phase (Eq 1), assuming no cell death during the eclipse phase, is given by *R*_0_ = *βpT*_0_/(*δc*) [54,55]. We reparametrize using *β* = *R*_0_δ*c*/(*pT*_0_) and for parameters *R*_0_, *δ*, *p* and *V*_*0*_, we estimated both the population parameter value and the inter-individual variability. Since the viral load data was fitted on a log_10_ scale we estimated the initial viral load, *V*_*0*_, on a log_10_ scale, allowing log_10_ *V*_*0*_ to take a normal distribution in the population, so that *V*_0_ follows a lognormal distribution like the other parameters. For the innate immune response models (Eqs 2 and 3 plus 4, 5, or 6), we continued to use the fixed values of *k* and *c* selected for the target cell limited model (Eq 2) and in addition we fixed α = 2 d^-1^ and *s* = 1 d^-1^.

#### Model selection

Model selection was based on log likelihoods (natural logarithms) to compare different model structures and Wald tests for inclusion of covariates, as provided by Monolix. Comparisons between nested models were performed using the log likelihood ratio test (LLRT).

To assess the effect of inoculum dose and viral strain we allowed for model parameters to depend on those two covariates (dose and strain) using the following standard procedure. Each possible covariate structure was added to the model one at a time and the parameter estimation algorithm was run for each. The possible covariate structures include relationships between each fitted parameter and either inoculum dose or viral strain. Inoculum dose was included as a continuous covariate. For a lognormally distributed parameter *θ* and the median value for individuals receiving inoculum dose *D* (log_10_ PFU) is given by: $\bar{\theta }=\theta_{pop}e^{B_{\theta }D}$ where the population parameter value θ_pop_ and the covariate coefficient B_θ_ are to be estimated. Viral strain was included as a categorical covariate. For a lognormally distributed parameter *θ* the median value is given by $\bar{\theta }=\theta_{pop}e^{S_{\theta }I_{PR}}$ where $I_{PR}$ is an indicator function, equal to 1 if individual is infected with PR ZIKV and equal to 0 otherwise, and as above θ_pop_ and S_θ_ are to be estimated.

We selected covariate relationships to incorporate into our model structure based on both statistical significance and model fit. A covariate relationship is included in the model structure if it both has a significant p-value (at a threshold of 0.05 after correction for multiple testing) by the Wald test as provided by Monolix and provides at least as good a log likelihood as the model without the relationship. If more than one covariate relationship fulfilled these criteria, the one with the lowest *p*-value was selected to be incorporated into the model first. After a covariate relationship was incorporated under these criteria, the remaining possible relationships were again retested on top of the incorporated relationships and this iterative procedure was repeated until no covariate relationships were supported statistically. Note that we did not fix the value of *B*_θ_ for any of the covariate relationships but allowed them to be estimated each time the fitting algorithm is run.

#### Model assessment

We visually inspected model fits to data at the individual level by obtaining predicted viral kinetics from estimated individual parameters and plotting these with the observed data measurements. At a population level, we used visual predictive checks (VPCs), whereby parameter sets are repeatedly selected at random (and independently) from the estimated distributions and predicted viral load dynamics are recorded for comparison with observed data. The range of these predicted viral kinetics are then plotted alongside the aggregated observed data measurements.

## Supporting information

S1 Text Models incorporating immune control of plasma viremia. (PDF)

S1 Table Observed plasma Zika viral loads used in this study, in log10 RNA copies per ml at days post infection (dpi), as reported by Aid et al. [9]. Where viral RNA was undetectable in a sample it is indicated at the limit of detection of the assay, 10^2^ RNA copies/ml.
(PDF)

S2 Table Results from adding covariate relationships to the target cell limited model (Eq 1). Only a covariate relationship been inoculum dose and initial plasma viral load *V*(0) is accepted and included in the model structure. Note that the *p*-values shown here are as provided by Monolix and are not corrected for multiple testing.
(PDF)

S3 Table Model fitting results from the target cell limited model and innate immune models. The value of τ shown is the one that was found to provide the maximum likelihood. The *p*-value shown is from a log likelihood ratio test against the target cell limited model with three degrees of freedom for the median and variability on the parameter γ and the fixed values of τ tested.
(PDF)

S4 Table Estimated population parameter values from fitting the innate immune model with reduced viral production (Eq 2) to viral load data from all animals using a non-linear mixed effects model. Relative standard errors are shown in parentheses. An explicit covariate relationship between inoculum dose and initial viral load *V*_*0*_ is incorporated, with the median population estimate for log_10_ *V*_0_ at each inoculum dose shown in italics. Fixed parameters used in the model fit are: *k* = 8 d^-1^, *c* = 10 d^-1^, *s* = 1 d^-1^, α = 2 d^-1^, *T*(0) = 10^5^ ml^-1^, each with no variability.
(PDF)

S5 Table Results from adding covariate relationships to the innate immune model with reduced viral production rate (Eq 2). Note that the *p*-values shown here are as provided by Monolix and are not corrected for multiple testing. The covariate relationship between inoculum dose and τ fulfils both the log likelihood and Wald test criteria (see methods) and so is accepted. No additional covariate relationships on top of this are accepted.
(PDF)

S6 Table Estimated population parameter values from fitting the innate immune model with reduced viral production (Eq 2) to viral load data from all animals using a non-linear mixed effects model. Relative standard errors are shown in parentheses. Explicit covariate relationships between inoculum dose and initial viral load *V*_*0*_ and between inoculum dose and delay to immune response τ are included, and the median population values of these parameters at each dose are shown in italics. Fixed parameters used in the model fit are: *k* = 8 d^-1^, *c* = 10 d^-1^, *s* = 1 d^-1^, α = 2 d^-1^, *T*(0) = 10^5^ ml^-1^, each with no variability.
(PDF)

S7 Table Results from adding covariate relationships to the viral interference model (Eq 3). No covariate relationships fulfil both criteria (log likelihood and *p*-value, see methods) to be included in the model. Note that the *p*-values shown here are as provided by Monolix and are not corrected for multiple testing. Given 11 tested covariate relationships, the significance threshold is 0.05/11 = 0.0045, and our testing criteria (see methods) require a covariate relationship with a significant Wald test and a log likelihood at least as good as the model without the added covariate relationship.
(PDF)

S1 Fig The log-likelihood of the target cell limited model (Eq 1) fit to plasma VL data from all 28 animals simultaneously, with different fixed values of *k* (the transition rate from the eclipse phase to productively infected) and *c* (the viral clearance rate). At each pair of values for *k* and *c* the model fitting algorithm is repeated 100 times with different initial guesses for the fitted parameters and different random seeds for the algorithm. Left: the log-likelihood from each repeated data fitting is shown by dots (visible in the inset), and the median is shown by the line. The inset shows the same data, focused on those fits providing the maximum log-likelihoods. Right: the median log-likelihood for each pair of values of *k* and *c*, colored by value. Those pairs of *k* and *c* which provide a median log-likelihood within 2 points of the maximum median log-likelihood are outlined and colored white.
(PDF)

S2 Fig Estimated population parameter values at each pair of fixed *c* and *k* that provide a median log- likelihood within 2 points of the maximum median log-likelihood. Results from each implementation of the fitting algorithm (as described in SF1) are shown by markers, medians are shown by horizontal bars and interquartile ranges are shown by vertical lines (often not visible due to tightly distributed estimates). The 100 implementations of the fitting algorithm shown here are each from different initial parameter guesses and random seeds.
(PDF)

S3 Fig Relationships between individual estimated parameters and inoculum dose. Individual estimated parameters are derived from the population fit of the target cell limited model (Eq 1) with fixed *k* = 8 d^-1^ and fixed *c* = 10 d^-1^, without any explicit inclusion of covariate structures between inoculum and parameter value. Correlations between parameters and inoculum dose are assessed via the Pearson correlation, with *p*-value (N.S. indicates non-significant relationship) and correlation coefficient shown above each panel. Where the correlation is statistically significant after Bonferroni correction the linear regression line is shown. Markers for individual animals are colored by the viral strain (BR: green triangles, PR: purple circles).
(PDF)

S4 Fig Relationships between individual estimated parameters and viral strain. Individual estimated parameters are derived from the population fit of the target cell limited model (Eq 1) with fixed *k* = 8 d^-1^ and fixed *c* = 10 d^-1^. Differences between parameters by viral strain are assessed by the Mann Whitney U test, with *p*-value (N.S. indicates non-significant relationship) shown in each panel. Markers for individual animals show the inoculum dose (10^3^ PFU: light blue triangles, 10^4^ PFU: dark blue squares, 10^5^ PFU: orange pentagons, 10^6^ PFU: red hexagons) and the horizontal black line represents the median parameter value for each group.
(PDF)

S5 Fig Visual predictive checks for the fit of the target cell limited model (Eq 1) with fixed *k* = 8 d^-1^ and fixed *c* = 10 d^-1^ and with a dose-dependency in log_10_ *V*_*0*_ explicitly incorporated (Table 1). 100,000 repeated random parameter sets were selected from the estimated parameter distributions for each inoculum dose, and predicted viral loads given these parameter values were recorded. Black lines show median predicted viral load, grey shaded region shows 2.5^th^– 97.5^th^ percentiles of predicted viral loads and black points indicate experimentally observed viral loads. The limit of detection of the experimental assay is shown with a horizontal dashed line and where experimental measurements failed to detect ZIKV in a sample it is shown with an open marker at this limit of detection.
(PDF)

S6 Fig Estimated parameter distributions from the target cell limited model (Eq 1) with fixed *k* = 8 d^-1^ and fixed *c* = 10 d^-1^ and with a dose-dependency in log_10_ *V*_*0*_ explicitly incorporated (Table 1). Inoculum dose indicated by color where relevant (light blue = 10^3^ PFU, dark blue = 10^4^ PFU, orange = 10^5^ PFU, red = 10^6^ PFU).
(PDF)

S7 Fig Relationships between individual estimated parameters and inoculum dose, with individual estimated parameters from the target cell limited model (Eq 1) with fixed *k* = 8 d^-1^ and fixed *c* = 10 d^-1^ and with a dose-dependency in log_10_ *V*_*0*_ explicitly incorporated (Table 1). Correlations between parameters and inoculum dose are assessed via the Pearson correlation, and where this is significant after Bonferroni correction the linear regression line is shown (dashed) and the *p*-value is shown above the panel. Markers for individual animals are colored by the viral strain (BR: green triangles, PR: purple circles).
(PDF)

S8 Fig Relationships between individual estimated parameters and viral strain, with individual estimated parameters from the target cell limited model (Eq 1) with fixed *k* = 8 d^-1^ and fixed *c* = 10 d^-1^ and with a dose-dependency in log_10_ *V*_*0*_ explicitly incorporated (Table 1). Differences between parameters by viral strain are assessed by the Mann Whitney U test, and no significant relationships after Bonferroni correction are observed. Markers for individual animals are colored by the viral strain (BR: green triangles, PR: purple circles).
(PDF)

S9 Fig Predicted viral loads for each individual animal from the fit of the target cell limited model (Eq 1) with fixed *k* = 8 d^-1^ and fixed *c* = 10 d^-1^ and with a dose-dependency in log_10_ *V*_*0*_ explicitly incorporated (Table 1). Color and marker shape indicate inoculum strain (BR: green triangles, PR: purple circles) and inoculum dose is indicated top left of each panel. Observed VLs are shown by markers and model prediction is shown by the solid line. The limit of detection of the experimental assay is shown by the horizontal dashed line and where ZIKV is not detectable in a sample it is shown with an open marker at this value.
(PDF)

S10 Fig The log likelihood from model fits where the population distribution of δ is a scaled logit-normal, so it is only able to take values up to a maximum, rather than a lognormal as for other parameters. Model fits are of the target cell limited model (Eq 1) with fixed *k* = 8 d^-1^ and fixed *c* = 10 d^-1^ and with a dose-dependency in log_10_ *V*_*0*_ explicitly incorporated. Each circle represents the output from a repeated fit with randomly selected initial parameter guesses and random seeds. The horizontal line shows the log-likelihood from the model fit with lognormally distributed δ.
(PDF)

S11 Fig Visual predictive checks for the fit of the innate immune model with reduced viral production rate (Eq 2) with fixed *k* = 8 d^-1^, fixed *c* = 10 d^-1^, fixed *s* = 1 d^-1^ and fixed α = 2 d^-1^, and with a dose-dependency in log_10_ *V*_*0*_ explicitly incorporated (S4 Table). 100,000 repeated random parameter sets were selected from the estimated parameter distributions for each inoculum dose, and predicted viral loads given these parameter values were recorded. Black lines show median predicted viral load, grey shaded region shows 2.5^th^– 97.5^th^ percentiles of predicted viral loads and black points indicate experimentally observed viral loads. The limit of detection of the experimental assay is shown with a horizontal dashed line and where experimental measurements failed to detect ZIKV in a sample it is shown with an open marker at this limit of detection.
(PDF)

S12 Fig Estimated parameter distributions from the innate immune model with reduced viral production rate (Eq 2) with fixed *k* = 8 d^-1^, fixed *c* = 10 d^-1^, fixed *s* = 1 d^-1^ and fixed α = 2 d^-1^, and with a dose-dependency in log_10_ *V*_*0*_ explicitly incorporated (S4 Table). Inoculum dose indicated by color where relevant (light blue = 10^3^ PFU, dark blue = 10^4^ PFU, orange = 10^5^ PFU, red = 10^6^ PFU).
(PDF)

S13 Fig Relationships between individual estimated parameters and inoculum dose, with individual estimated parameters from the innate immune model with reduced viral production rate (Eq 2) with fixed *k* = 8 d^-1^, fixed *c* = 10 d^-1^, fixed *s* = 1 d^-1^ and fixed α = 2 d^-1^, and with a dose-dependency in log_10_ *V*_*0*_ explicitly incorporated (S4 Table). Correlations between parameters and inoculum dose are assessed via the Pearson correlation, and where this is significant after Bonferroni correction the linear regression line is shown (dashed) and the *p*-value is shown above the panel. Markers for individual animals are colored by the viral strain (BR: green triangles, PR: purple circles).
(PDF)

S14 Fig Relationships between individual estimated parameters and viral strain, with individual estimated parameters from the innate immune model with reduced viral production rate (Eq 2) with fixed *k* = 8 d^-1^, fixed *c* = 10 d^-1^, fixed *s* = 1 d^-1^ and fixed α = 2 d^-1^, and with a dose-dependency in log_10_ *V*_*0*_ explicitly incorporated (S4 Table). Differences between parameters by viral strain are assessed by the Mann Whitney U test, and no significant relationships after Bonferroni correction are observed. Markers for individual animals are colored by the viral strain (BR: green triangles, PR: purple circles).
(PDF)

S15 Fig Predicted viral loads for each individual animal from the fit of the innate immune model with reduced viral production rate (Eq 2) with fixed *k* = 8 d^-1^, fixed *c* = 10 d^-1^, fixed *s* = 1 d^-1^ and fixed α = 2 d^-1^, and with a dose-dependency in log_10_ *V*_*0*_ explicitly incorporated (S4 Table). Color and marker shape indicate inoculum strain (BR: green triangles, PR: purple circles) and inoculum dose is indicated top left of each panel. Observed VLs are shown by markers and model prediction is shown by the solid line. The limit of detection of the experimental assay is shown by the horizontal dashed line and where ZIKV is not detectable in a sample it is shown with an open marker at this value.
(PDF)

S16 Fig Estimated log likelihood and population parameters (Table 1) of the innate immune model with reduced viral production rate (Eq 2) with fixed *k* = 8 d^-1^, fixed *c* = 10 d^-1^, fixed *s* = 1 d^-1^ and fixed α as indicated on the horizontal axis. Each circle represents the model fit from one implementation of the fitting algorithm with randomly selected initial guesses and random seed. In the likelihood panel, the horizontal line shows the maximum likelihood with fixed α = 2 d^-1^ (S9 Fig and Table 1). The p-values (N.S denotes non-significance after Bonferroni correction) above each panel are from a Friedman test for repeated measurements.
(PDF)

S17 Fig Visual predictive checks for the fit of the innate immune model with reduced viral production rate (Eq 2) with fixed *k* = 8 d^-1^, fixed *c* = 10 d^-1^, fixed *s* = 1 d^-1^ and fixed α = 2 d^-1^, and with dose-dependencies in log_10_ *V*_*0*_ and in τ explicitly incorporated (S6 Table). 100,000 repeated random parameter sets were selected from the estimated parameter distributions for each inoculum dose, and predicted viral loads given these parameter values were recorded. Black lines show median predicted viral load, grey shaded region shows 2.5^th^– 97.5^th^ percentiles of predicted viral loads and black points indicate experimentally observed viral loads. The limit of detection of the experimental assay is shown with a horizontal dashed line and where experimental measurements failed to detect ZIKV in a sample it is shown with an open marker at this limit of detection.
(PDF)

S18 Fig Estimated parameter distributions from the innate immune model with reduced viral production rate (Eq 2) with fixed *k* = 8 d^-1^, fixed *c* = 10 d^-1^, fixed *s* = 1 d^-1^ and fixed α = 2 d^-1^, and with dose-dependencies in log_10_ *V*_*0*_ and in τ explicitly incorporated (S6 Table). Inoculum dose indicated by color where relevant (light blue = 10^3^ PFU, dark blue = 10^4^ PFU, orange = 10^5^ PFU, red = 10^6^ PFU).
(PDF)

S19 Fig Relationships between individual estimated parameters and inoculum dose, with individual estimated parameters from the innate immune model with reduced viral production rate (Eq 2) with fixed *k* = 8 d^-1^, fixed *c* = 10 d^-1^, fixed *s* = 1 d^-1^ and fixed α = 2 d^-1^, and with dose-dependencies in log_10_ *V*_*0*_ and in τ explicitly incorporated (S6 Table). Correlations between parameters and inoculum dose are assessed via the Pearson correlation, and where this is significant after Bonferroni correction the linear regression line is shown (dashed) and the *p*-value is shown above the panel. Markers for individual animals are colored by the viral strain (BR: green triangles, PR: purple circles).
(PDF)

S20 Fig Relationships between individual estimated parameters and viral strain, with individual estimated parameters from the innate immune model with reduced viral production rate (Eq 2) with fixed *k* = 8 d^-1^, fixed *c* = 10 d^-1^, fixed *s* = 1 d^-1^ and fixed α = 2 d^-1^, and with dose-dependencies in log_10_ *V*_*0*_ and in τ explicitly incorporated (S6 Table). Differences between parameters by viral strain are assessed by the Mann Whitney U test, and no significant relationships after Bonferroni correction are observed. Markers for individual animals are colored by the viral strain (BR: green triangles, PR: purple circles).
(PDF)

S21 Fig Predicted viral loads for each individual animal from the fit of the innate immune model with reduced viral production rate (Eq 2) with fixed *k* = 8 d^-1^, fixed *c* = 10 d^-1^, fixed *s* = 1 d^-1^ and fixed α = 2 d^-1^, and with dose-dependencies in log_10_ *V*_*0*_ and in τ explicitly incorporated (S6 Table). Color and marker shape indicate inoculum strain (BR: green triangles, PR: purple circles) and inoculum dose is indicated top left of each panel. Observed VLs are shown by markers and model prediction is shown by the solid line. The limit of detection of the experimental assay is shown by the horizontal dashed line and where ZIKV is not detectable in a sample it is shown with an open marker at this value.
(PDF)

S22 Fig The relationship between viral load *V* (x-axis) and viral interference *g*(*V*) in the viral interference model (Eq 3). (PDF)

S23 Fig Maximum log likelihoods, colored by value, found from fitting the viral interference model (Eq 3) to observed viral load data with different values of *K* (the half maximal response parameter) and *n* (the Hill coefficient). Those fits which are indistinguishable by log likelihood, within 2 points of the maximum, are highlighted in white.
(PDF)

S24 Fig Visual predictive checks for the fit of the viral interference model (Eq 3) with fixed *k* = 8 d^-1^, fixed *c* = 10 d^-1^, fixed *s* = 1 d^-1^, fixed α = 2 d^-1^, fixed *K* = 10^3^ and fixed *n* = 0.25, and with a dose-dependency in log_10_ *V*_*0*_ explicitly incorporated (Table 1). 100,000 repeated random parameter sets were selected from the estimated parameter distributions for each inoculum dose, and predicted viral loads given these parameter values were recorded. Black lines show median predicted viral load, grey shaded region shows 2.5^th^– 97.5^th^ percentiles of predicted viral loads and black points indicate experimentally observed viral loads. The limit of detection of the experimental assay is shown with a horizontal dashed line and where experimental measurements failed to detect ZIKV in a sample it is shown with an open marker at this limit of detection.
(PDF)

S25 Fig Estimated parameter distributions from the viral interference model (Eq 3) with fixed *k* = 8 d^-1^, fixed *c* = 10 d^-1^, fixed *s* = 1 d^-1^, fixed α = 2 d^-1^, fixed *K* = 10^3^ and fixed *n* = 0.25, and with a dose-dependency in log_10_ *V*_*0*_ explicitly incorporated (Table 1). Inoculum dose indicated by color where relevant (light blue = 10^3^ PFU, dark blue = 10^4^ PFU, orange = 10^5^ PFU, red = 10^6^ PFU).
(PDF)

S26 Fig Relationships between individual estimated parameters and inoculum dose, with individual estimated parameters from the viral interference model (Eq 3) with fixed *k* = 8 d^-1^, fixed *c* = 10 d^-1^, fixed *s* = 1 d^-1^, fixed α = 2 d^-1^, fixed *K* = 10^3^ and fixed *n* = 0.25, and with a dose-dependency in log_10_ *V*_*0*_ explicitly incorporated (Table 1). Correlations between parameters and inoculum dose are assessed via the Pearson correlation, and where this is significant after Bonferroni correction the linear regression line is shown (dashed) and the *p*-value is shown above the panel. Markers for individual animals are colored by the viral strain (BR: green triangles, PR: purple circles).
(PDF)

S27 Fig Relationships between individual estimated parameters and viral strain, with individual estimated parameters from the viral interference model (Eq 3) with fixed *k* = 8 d^-1^, fixed *c* = 10 d^-1^, fixed *s* = 1 d^-1^, fixed α = 2 d^-1^, fixed *K* = 10^3^ and fixed *n* = 0.25, and with a dose-dependency in log_10_ *V*_*0*_ explicitly incorporated (Table 1). Differences between parameters by viral strain are assessed by the Mann Whitney U test, and no significant relationships after Bonferroni correction are observed. Markers for individual animals are colored by the viral strain (BR: green triangles, PR: purple circles).
(PDF)

S28 Fig Predicted viral loads for each individual animal from the viral interference model (Eq 3) with fixed *k* = 8 d^-1^, fixed *c* = 10 d^-1^, fixed *s* = 1 d^-1^, fixed α = 2 d^-1^, fixed *K* = 10^3^ and fixed *n* = 0.25, and with a dose-dependency in log_10_ *V*_*0*_ explicitly incorporated (Table 1). Color and marker shape indicate inoculum strain (BR: green triangles, PR: purple circles) and inoculum dose is indicated top left of each panel. Observed VLs are shown by markers and model prediction is shown by the solid line. The limit of detection of the experimental assay is shown by the horizontal dashed line and where ZIKV is not detectable in a sample it is shown with an open marker at this value.
(PDF)

S29 FigImmune response summary quantities by inoculum dose for the viral interference model (Eq 3, Table 1).Top: the area under the curve (AUC) of the immune-response-restricted viral production rate ($\hat{p}(t)$) normalized by the estimated viral production rate in the absence of immune response (*p*) for each animal. Middle: The maximum effect of immune response on viral production rate, with 1 representing complete control of viral production, 0 representing no restriction of viral production. Bottom: the AUC of the total viral production, given by the immune restricted viral production rate $\hat{p}(t)$ multiplied by the productive infected cell concentration *I*_2_(t). In each panel, the p-value shown is from a linear regression and where this is statistically significant (p < 0.05) the linear regression line is shown.(PDF)

S30 Fig The mean predicted viral load (left axis, black) and mean predicted target cell concentration normalized by *T*(0) (purple, right axis) at each dose group, from the innate immune model with restricted viral production and viral interference (Eq 3, Table 1). (PDF)

S31 Fig Viral load (VL) characteristics from 100 simulated viral dynamics profiles with model parameters selected from the fit of the viral interference model (Eq 3, Table 1). Correlations between inoculum dose and viral characteristic are assessed via a Pearson correlation, with *p*-value shown in each panel. Where this relationship is found to be significant at the α = 0.05 level after Bonferroni correction for multiple testing (*m* = 6) the linear regression line is shown in the panel. The VL AUC and downslopes are calculated as for the observed viral loads, described in Methods.
(PDF)
