# Supplementary material for: Zika virus dynamics: Effects of inoculum dose, the innate immune response and viral interference
Source: PLoS Comput Biol. 2021 Jan 20;17(1):e1008564. doi: 10.1371/journal.pcbi.1008564 (PMC7817008; doi:10.1371/journal.pcbi.1008564)
Supplement: S1 Text — (PDF) [file pcbi.1008564.s001.pdf]

Supplementary Material

Models incorporating immune control of plasma viremia

We incorporated an innate immune response into the target cell limited model by including a model of a generic innate immune response, X. As in the work of Baccam et al. [14] on the innate response to influenza infection, we assumed this generic innate immune response is produced at a rate proportional to the concentration of productively infected cells, after a time delay τ, and decays at a constant rate:

$$dX/dt = sI_2\,(t - \tau) - \alpha X \qquad \text{(Eq. S1)}$$

Without loss of generality and for the sake of identifiability we set the coefficient of production of the innate response, s, equal to 1 such that X is in units of daily production.

The effect of innate immune response is then modelled in one of three ways, with the strength of the immune effect described with an additional parameter γ.

(i) The viral infectivity is reduced: $\hat{\beta} = \beta/(1 + \gamma X)$ (Eq. S2)

(ii) The death rate of productively infected cells in increased: $\hat{\delta} = \delta(1 + \gamma X)$ (Eq. S3)

(iii) The rate of viral production from infected cells is reduced: $\hat{p} = p/(1 + \gamma X)$ (Eq. S4)

For the initial model fitting the degradation rate α was fixed to 2 d^{-1} and a number of different fixed values of τ, the delay before immune response initiation, were tested without any inter-individual variability. The distribution of γ, the strength of the immune response, was allowed to be freely fitted. Each of these innate immune response models was statistically supported as providing a better fit to the data than the target cell limited model (Supplementary Table 3). The model with innate immune response reducing viral production provided the best fit to the data by log likelihood and this model was therefore selected to be analysed further.
