# Supplementary material for: Zika virus dynamics: Effects of inoculum dose, the innate immune response and viral interference
Source: PLoS Comput Biol. 2021 Jan 20;17(1):e1008564. doi: 10.1371/journal.pcbi.1008564 (PMC7817008; doi:10.1371/journal.pcbi.1008564)
Supplement: S1 Table — Where viral RNA was undetectable in a sample it is indicated at the limit of detection of the assay, 102 RNA copies/ml. (PDF) [file pcbi.1008564.s002.pdf]

Supplementary Table 1

Observed plasma Zika viral loads used in this study, in $\log_{10}$ RNA copies per ml at days post infection (dpi), as reported by Aid et al. [9]. Where viral RNA was undetectable in a sample it is indicated at the limit of detection of the assay, 10^2 RNA copies/ml.

Macaque ID	Inoculum dose ($\log_{10}$ PFU)	Viral strain	1dpi	2dpi	3dpi	4dpi	5dpi	6dpi	7dpi	10dpi	14dpi	21dpi
1103204	6	BR	5.26	6.21	5.96	5.14	4.26	3.05	2.46	2	2	2
1002170	6	BR	5.22	6.22	4.88	3.22	2.86	2	2	2	2	2
R567	5	BR	4.04	5.71	6.31	5.03	3.99	3.50	3.47	2	2	2
1106194	5	BR	4.45	5.57	6.02	4.22	2.48	2	2	2	2	2
1205200	4	BR	4.03	5.78	6.48	6.24	5.04	3.15	2.62	2	2	2
1104228	4	BR	3.99	4.93	5.77	5.71	3.48	2	2	2	2	2
R590	3	BR	3.22	4.46	4.90	5.60	5.75	4.32	4.57	2	2	2
1104212	3	BR	2.33	3.27	4.30	5.70	5.47	3.37	2	2	2	2
1105028	6	PR	5.40	6.71	5.93	4.08	2	2	-	2	2	2
5265	6	PR	5.41	6.27	6.10	5.01	3.47	2.96	2	2	2	2
4851	5	PR	4.25	5.85	3.19	5.14	4.25	2	2	2	2	2
1105012	5	PR	4.55	6.15	6.19	5.22	3.58	2.35	2	2	2	2
R588	4	PR	2.78	5.62	5.85	5.44	4.48	2.48	2	2	2	2
1105186	4	PR	3.14	4.45	5.18	4.46	2.79	2	2	2	2	2
1104224	3	PR	2	3.13	4.69	5.65	4.44	3.89	2	2	2	2
5266	3	PR	2.46	4.06	4.39	5.31	5.84	4.96	3.63	2	2	2
T566	3	BR	2	3.52	4.97	5.38	6.42	5.44	2.82	-	-	-
T567	3	BR	2.16	3.58	4.85	5.56	6.24	4.64	2.88	-	-	-
T568	3	BR	2.37	4.26	5.82	6.04	6.23	5.12	3.10	-	-	-
T569	3	BR	2.08	3.10	5.25	5.97	7.00	5.86	3.72	-	-	-
T528	3	BR	3.72	4.53	5.48	6.29	5.69	3.01	2	-	-	-
T529	3	BR	2	3.77	4.90	5.51	4.42	2	2	-	-	-
T530	3	BR	2	3.87	4.54	5.91	6.00	3.69	2	-	-	-
T545	3	BR	3.73	5.01	6.07	5.91	4.29	3.54	2	-	-	-
T535	3	PR	3.09	4.39	5.33	6.11	5.86	3.40	2	-	-	-
T536	3	PR	2.83	3.82	5.21	4.90	4.73	3.14	2	-	-	-
T537	3	PR	2.59	4.39	5.06	5.49	5.31	4.19	2	-	-	-
T546	3	PR	2.41	5.00	5.52	5.93	5.36	3.97	2	-	-	-
