# Supplementary material for: Zika virus dynamics: Effects of inoculum dose, the innate immune response and viral interference
Source: PLoS Comput Biol. 2021 Jan 20;17(1):e1008564. doi: 10.1371/journal.pcbi.1008564 (PMC7817008; doi:10.1371/journal.pcbi.1008564)
Supplement: S2 Table — Only a covariate relationship been inoculum dose and initial plasma viral load V(0) is accepted and included in the model structure. Note that the p-values shown here are as provided by Monolix and are not corrected for multiple testing. (PDF) [file pcbi.1008564.s003.pdf]

Supplementary Table 2

Results from adding covariate relationships to the target cell limited model (Eq. 1). Only a covariate relationship been inoculum dose and initial plasma viral load $V(0)$ is accepted and included in the model structure. Note that the p-values shown here are as provided by Monolix and are not corrected for multiple testing.

**base model**	**covariate relationship added**	**log likelihood**	**covariate coefficient**	**p-value (Wald test)**
Target cell limited model with no covariates	None	-194.3	-	-
	Inoculum dose on R_0	-194.3	0.022	0.48
	Inoculum dose on δ	-194.3	-0.064	0.3
	Inoculum dose on p	-194.1	0.158	0.25
	Inoculum dose on $\log_{10} V(0)$	-169.2	1.00	$< 10^{-10}$
	Viral strain on R_0	-194.3	0.061	0.26
	Viral strain on δ	-194.0	0.135	0.29
	Viral strain on p	-194.3	-0.254	0.42
	Viral strain on $\log_{10} V(0)$	-194.8	0.183	0.7
Target cell limited model with dose-$V(0)$ covariate	None	-169.2	-	-
	Inoculum dose on R_0	-168.3	-0.043	0.2
	Inoculum dose on δ	-167.4	-0.13	0.09
	Inoculum dose on p	-168.2	0.204	0.14
	Viral strain on R_0	-169.0	0.036	0.36
	Viral strain on δ	-168.6	0.090	0.39
	Viral strain on p	-169.3	-0.257	0.42
	Viral strain on $\log_{10} V(0)$	-169.4	-0.021	0.91
