# Supplementary material for: Zika virus dynamics: Effects of inoculum dose, the innate immune response and viral interference
Source: PLoS Comput Biol. 2021 Jan 20;17(1):e1008564. doi: 10.1371/journal.pcbi.1008564 (PMC7817008; doi:10.1371/journal.pcbi.1008564)
Supplement: S3 Table — The value of τ shown is the one that was found to provide the maximum likelihood. The p-value shown is from a log likelihood ratio test against the target cell limited model with three degrees of freedom for the median and variability on the parameter γ and the fixed values of τ tested. (PDF) [file pcbi.1008564.s004.pdf]

Supplementary Table 3

Model fitting results from the target cell limited model and innate immune models. The value of τ shown is the one that was found to provide the maximum likelihood. The *p*-value shown is from a log likelihood ratio test against the target cell limited model with three degrees of freedom for the median and variability on the parameter γ and the fixed values of τ tested.

**Model**	**τ (d)**	**Log likelihood**	**LLRT p value**
Target cell limited (Eq. 1)	-	-169.2	-
Reduced viral infectivity	2	-154.4	$<10^{-5}$
Enhanced clearance of infected cells	2	-154.7	$<10^{-5}$
Reduced viral production	3	-147.0	$<10^{-8}$
