# Supplementary material for: Zika virus dynamics: Effects of inoculum dose, the innate immune response and viral interference
Source: PLoS Comput Biol. 2021 Jan 20;17(1):e1008564. doi: 10.1371/journal.pcbi.1008564 (PMC7817008; doi:10.1371/journal.pcbi.1008564)
Supplement: S4 Table — Relative standard errors are shown in parentheses. An explicit covariate relationship between inoculum dose and initial viral load V0 is incorporated, with the median population estimate for log10 V0 at each inoculum dose shown in italics. Fixed parameters used in the model fit are: k = 8 d-1, c = 10 d-1, s = 1 d-1, α = 2 d-1, T(0) = 105 ml-1, each with no variability. (PDF) [file pcbi.1008564.s005.pdf]

Supplementary Table 4

Estimated population parameter values from fitting the innate immune model with reduced viral production (Eq. 2) to viral load data from all animals using a non-linear mixed effects model. Relative standard errors are shown in parentheses. An explicit covariate relationship between inoculum dose and initial viral load V_0 is incorporated, with the median population estimate for $\log_{10} V_0$ at each inoculum dose shown in italics. Fixed parameters used in the model fit are: $k = 8$ d^{-1}, $c = 10$ d^{-1}, $s = 1$ d^{-1}, $\alpha = 2$ d^{-1}, $T(0) = 10^5$ ml^{-1}, each with no variability.

**Parameter**	**Population estimate**		**Covariate coefficient**		***p*-value**	**Variability estimate**	
R_0	3.44	(12%)				0.0284	(888%)
δ	3.31 d^{-1}	(21%)				0.116	(103%)
p	637 d^{-1}	(24%)				0.536	(24%)
γ	3.36	(49%)				0.859	(52%)
τ	5.37 d	(5%)				0.239	(15%)
$\log_{10} V_0$	-1.00 ml^{-1}	(8%)	0.97	(8%)	$<10^{-10}$	0.341	(26%)
at 10^3 PFU	*1.91 ml^{-1}*						
at 10^4 PFU	*2.87 ml^{-1}*						
at 10^5 PFU	*3.84 ml^{-1}*						
at 10^6 PFU	*4.81 ml^{-1}*						
