# Supplementary material for: Zika virus dynamics: Effects of inoculum dose, the innate immune response and viral interference
Source: PLoS Comput Biol. 2021 Jan 20;17(1):e1008564. doi: 10.1371/journal.pcbi.1008564 (PMC7817008; doi:10.1371/journal.pcbi.1008564)
Supplement: S5 Table — Note that the p-values shown here are as provided by Monolix and are not corrected for multiple testing. The covariate relationship between inoculum dose and τ fulfils both the log likelihood and Wald test criteria (see methods) and so is accepted. No additional covariate relationships on top of this are accepted. (PDF) [file pcbi.1008564.s006.pdf]

Supplementary Table 5

Results from adding covariate relationships to the innate immune model with reduced viral production rate (Eq. 2). Note that the *p*-values shown here are as provided by Monolix and are not corrected for multiple testing. The covariate relationship between inoculum dose and τ fulfils both the log likelihood and Wald test criteria (see methods) and so is accepted. No additional covariate relationships on top of this are accepted.

Base model	covariate relationship added	log likelihood	covariate coefficient	p-value (Wald test)
Innate immune model with reduced viral production rate, including a dose-$V(0)$ covariate	None	-148.5	-	-
	Inoculum dose on R_0	-146.1	0.178	0.02
	Inoculum dose on δ	-148.8	0.044	0.46
	Inoculum dose on p	-148.4	0.078	0.54
	Inoculum dose on γ	-148.0	-1.52	0.12
	Inoculum dose on τ	-146.2	0.178	0.00035
	Viral strain on R_0	-148.9	0.033	0.55
	Viral strain on δ	-148.3	0.052	0.53
	Viral strain on p	-147.9	-0.361	0.16
	Viral strain on γ	-148.2	-0.57	0.64
	Viral strain on τ	-148.6	-0.076	0.48
	Viral strain on $\log_{10} V(0)$	-148.7	-0.024	0.89
Innate immune model with reduced viral production rate, including dose-$V(0)$ and dose-τ covariates	None	-146.2	-	-
	Inoculum dose on R_0	-144.1	0.136	0.044
	Inoculum dose on δ	-146.0	0.019	0.77
	Inoculum dose on p	-146.1	0.058	0.63
	Inoculum dose on γ	-146.0	0.705	0.55
	Viral strain on R_0	-146.3	-0.007	0.92
	Viral strain on δ	-146.5	0.122	0.19
	Viral strain on p	-145.1	-0.376	0.13
	Viral strain on γ	-145.5	0.107	0.9
	Viral strain on τ	-146.2	-0.157	0.15
	Viral strain on $\log_{10} V(0)$	-146.3	-0.004	0.82
