# Supplementary material for: Zika virus dynamics: Effects of inoculum dose, the innate immune response and viral interference
Source: PLoS Comput Biol. 2021 Jan 20;17(1):e1008564. doi: 10.1371/journal.pcbi.1008564 (PMC7817008; doi:10.1371/journal.pcbi.1008564)
Supplement: S6 Table — Relative standard errors are shown in parentheses. Explicit covariate relationships between inoculum dose and initial viral load V0 and between inoculum dose and delay to immune response τ are included, and the median population values of these parameters at each dose are shown in italics. Fixed parameters used in the model fit are: k = 8 d-1, c = 10 d-1, s = 1 d-1, α = 2 d-1, T(0) = 105 ml-1, each with no variability. (PDF) [file pcbi.1008564.s007.pdf]

Supplementary Table 6

Estimated population parameter values from fitting the innate immune model with reduced viral production (Eq. 2) to viral load data from all animals using a non-linear mixed effects model. Relative standard errors are shown in parentheses. Explicit covariate relationships between inoculum dose and initial viral load V_0 and between inoculum dose and delay to immune response τ are included, and the median population values of these parameters at each dose are shown in italics. Fixed parameters used in the model fit are: $k = 8$ d^{-1}, $c = 10$ d^{-1}, $s = 1$ d^{-1}, $\alpha = 2$ d^{-1}, $T(0) = 10^5$ ml^{-1}, each with no variability.

**Parameter**	**Population estimate**		**Covariate coefficient**		***p*-value**	**Variability estimate**	
R_0	3.77	(14%)				0.0343	(562%)
δ	2.77 d^{-1}	(19%)				0.104	(43%)
p	530 d^{-1}	(22%)				0.537	(25%)
γ	0.043	(59%)				0.877	(80%)
τ	2.02 d	(20%)	0.178	(28%)	0.00035	0.289	(15%)
at 10^3 PFU	*3.45 d*						
at 10^4 PFU	*4.12 d*						
at 10^5 PFU	*4.92 d*						
at 10^6 PFU	*5.88 d*						
$\log_{10} V_0$	-0.98 ml^{-1}	(33%)	0.96	(8%)	$<10^{-10}$	0.327	(30%)
at 10^3 PFU	*1.91 ml^{-1}*						
at 10^4 PFU	*2.87 ml^{-1}*						
at 10^5 PFU	*3.83 ml^{-1}*						
at 10^6 PFU	*4.80 ml^{-1}*						
