# Supplementary material for: Zika virus dynamics: Effects of inoculum dose, the innate immune response and viral interference
Source: PLoS Comput Biol. 2021 Jan 20;17(1):e1008564. doi: 10.1371/journal.pcbi.1008564 (PMC7817008; doi:10.1371/journal.pcbi.1008564)
Supplement: S7 Table — No covariate relationships fulfil both criteria (log likelihood and p-value, see methods) to be included in the model. Note that the p-values shown here are as provided by Monolix and are not corrected for multiple testing. Given 11 tested covariate relationships, the significance threshold is 0.05/11 = 0.0045, and our testing criteria (see methods) require a covariate relationship with a significant Wald test and a log likelihood at least as good as the model without the added covariate relationship. (PDF) [file pcbi.1008564.s008.pdf]

Supplementary Table 7

Results from adding covariate relationships to the viral interference model (Eq. 3). No covariate relationships fulfil both criteria (log likelihood and *p*-value, see methods) to be included in the model. Note that the *p*-values shown here are as provided by Monolix and are not corrected for multiple testing. Given 11 tested covariate relationships, the significance threshold is 0.05/11 = 0.0045, and our testing criteria (see methods) require a covariate relationship with a significant Wald test and a log likelihood at least as good as the model without the added covariate relationship.

**Base model**	**covariate relationship added**	**log likelihood**	**covariate coefficient**	**p-value (Wald test)**
Viral interference model including a dose-$V(0)$ covariate	None	-145.0	-	-
	Inoculum dose on R_0	-146.4	0.163	0.034
	Inoculum dose on δ	-149.9	0.057	0.33
	Inoculum dose on p	-144.6	0.14	0.27
	Inoculum dose on γ	-141.8	-0.927	0.016
	Inoculum dose on τ	-146.2	0.165	0.0005
	Viral strain on R_0	-149.5	0.024	0.7
	Viral strain on δ	-144.3	0.078	0.3
	Viral strain on p	-148.7	-0.424	0.14
	Viral strain on γ	-171.4	0.081	0.92
	Viral strain on τ	-149.3	-0.059	0.52
	Viral strain on $\log_{10} V(0)$	-149.2	-0.065	0.72
