# Supplementary material for: Zika virus dynamics: Effects of inoculum dose, the innate immune response and viral interference
Source: PLoS Comput Biol. 2021 Jan 20;17(1):e1008564. doi: 10.1371/journal.pcbi.1008564 (PMC7817008; doi:10.1371/journal.pcbi.1008564)
Supplement: S1 Fig — At each pair of values for k and c the model fitting algorithm is repeated 100 times with different initial guesses for the fitted parameters and different random seeds for the algorithm. Left: the log-likelihood from each repeated data fitting is shown by dots (visible in the inset), and the median is shown by the line. The inset shows the same data, focused on those fits providing the maximum log-likelihoods. Right: the median log-likelihood for each pair of values of k and c, colored by value. Those pairs of k and c which provide a median log-likelihood within 2 points of the maximum median log-likelihood are outlined and colored white. (PDF) [file pcbi.1008564.s009.pdf]

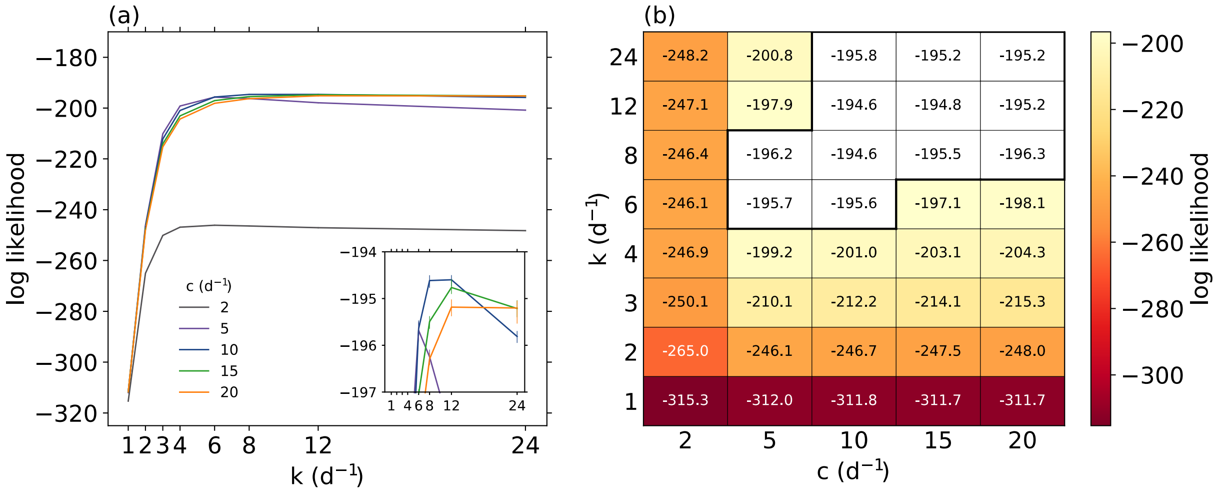

Supplementary Figure 1

The log-likelihood of the target cell limited model (Eq. 1) fit to plasma VL data from all 28 animals simultaneously, with different fixed values of *k* (the transition rate from the eclipse phase to productively infected) and *c* (the viral clearance rate). At each pair of values for *k* and *c* the model fitting algorithm is repeated 100 times with different initial guesses for the fitted parameters and different random seeds for the algorithm. Left: the log-likelihood from each repeated data fitting is shown by dots (visible in the inset), and the median is shown by the line. The inset shows the same data, focused on those fits providing the maximum log-likelihoods. Right: the median log-likelihood for each pair of values of *k* and *c*, colored by value. Those pairs of *k* and *c* which provide a median log-likelihood within 2 points of the maximum median log-likelihood are outlined and colored white.
