# Supplementary material for: Zika virus dynamics: Effects of inoculum dose, the innate immune response and viral interference
Source: PLoS Comput Biol. 2021 Jan 20;17(1):e1008564. doi: 10.1371/journal.pcbi.1008564 (PMC7817008; doi:10.1371/journal.pcbi.1008564)
Supplement: S2 Fig — Results from each implementation of the fitting algorithm (as described in SF1) are shown by markers, medians are shown by horizontal bars and interquartile ranges are shown by vertical lines (often not visible due to tightly distributed estimates). The 100 implementations of the fitting algorithm shown here are each from different initial parameter guesses and random seeds. (PDF) [file pcbi.1008564.s010.pdf]

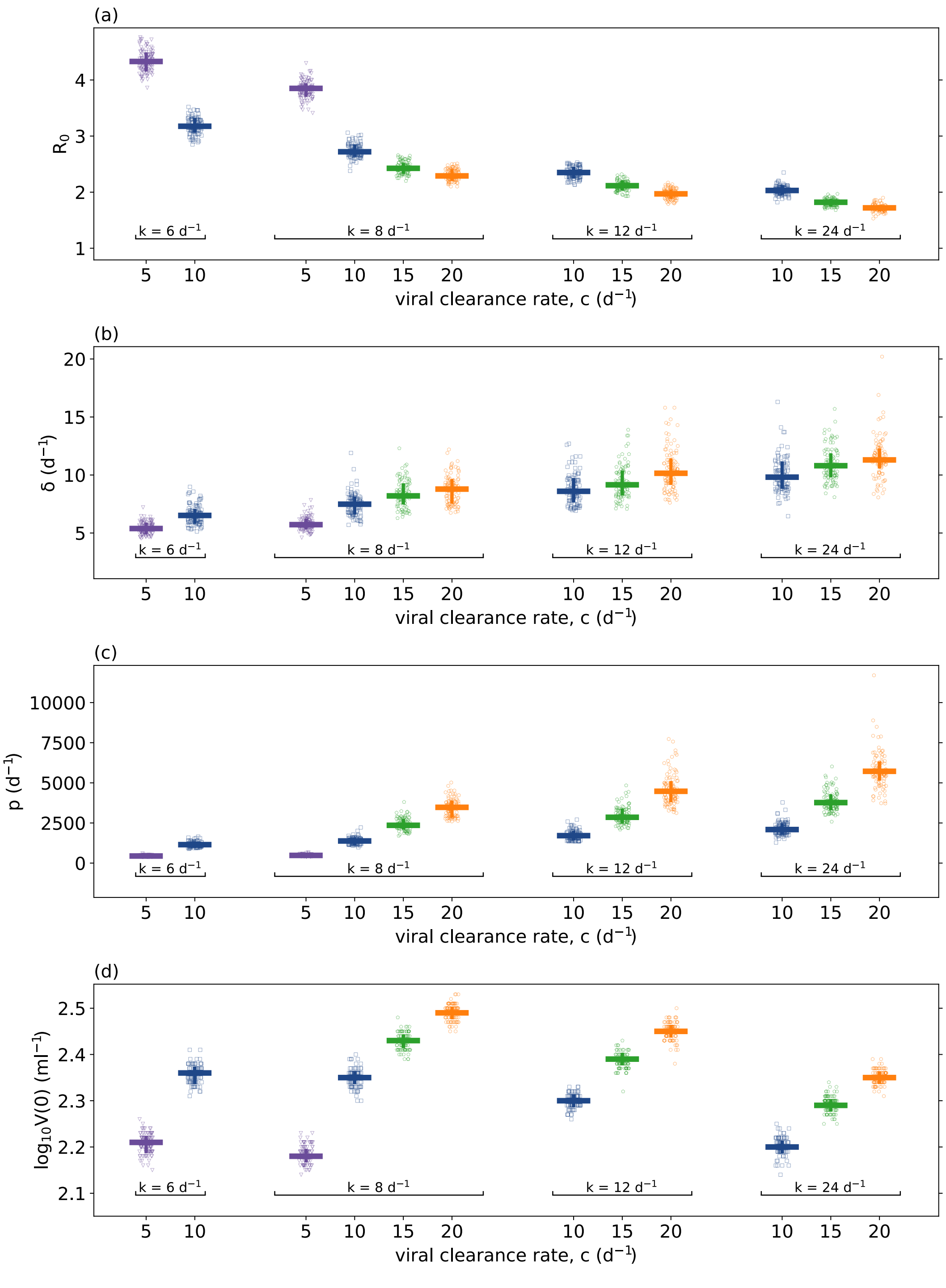

Supplementary Figure 2

Estimated population parameter values at each pair of fixed *c* and *k* that provide a median log-likelihood within 2 points of the maximum median log-likelihood. Results from each implementation of the fitting algorithm (as described in SF1) are shown by markers, medians are shown by horizontal bars and interquartile ranges are shown by vertical lines (often not visible due to tightly distributed estimates). The 100 implementations of the fitting algorithm shown here are each from different initial parameter guesses and random seeds.
