# Supplementary material for: Zika virus dynamics: Effects of inoculum dose, the innate immune response and viral interference
Source: PLoS Comput Biol. 2021 Jan 20;17(1):e1008564. doi: 10.1371/journal.pcbi.1008564 (PMC7817008; doi:10.1371/journal.pcbi.1008564)
Supplement: S3 Fig — Individual estimated parameters are derived from the population fit of the target cell limited model (Eq 1) with fixed k = 8 d-1 and fixed c = 10 d-1, without any explicit inclusion of covariate structures between inoculum and parameter value. Correlations between parameters and inoculum dose are assessed via the Pearson correlation, with p-value (N.S. indicates non-significant relationship) and correlation coefficient shown above each panel. Where the correlation is statistically significant after Bonferroni correction the linear regression line is shown. Markers for individual animals are colored by the viral strain (BR: green triangles, PR: purple circles). (PDF) [file pcbi.1008564.s011.pdf]

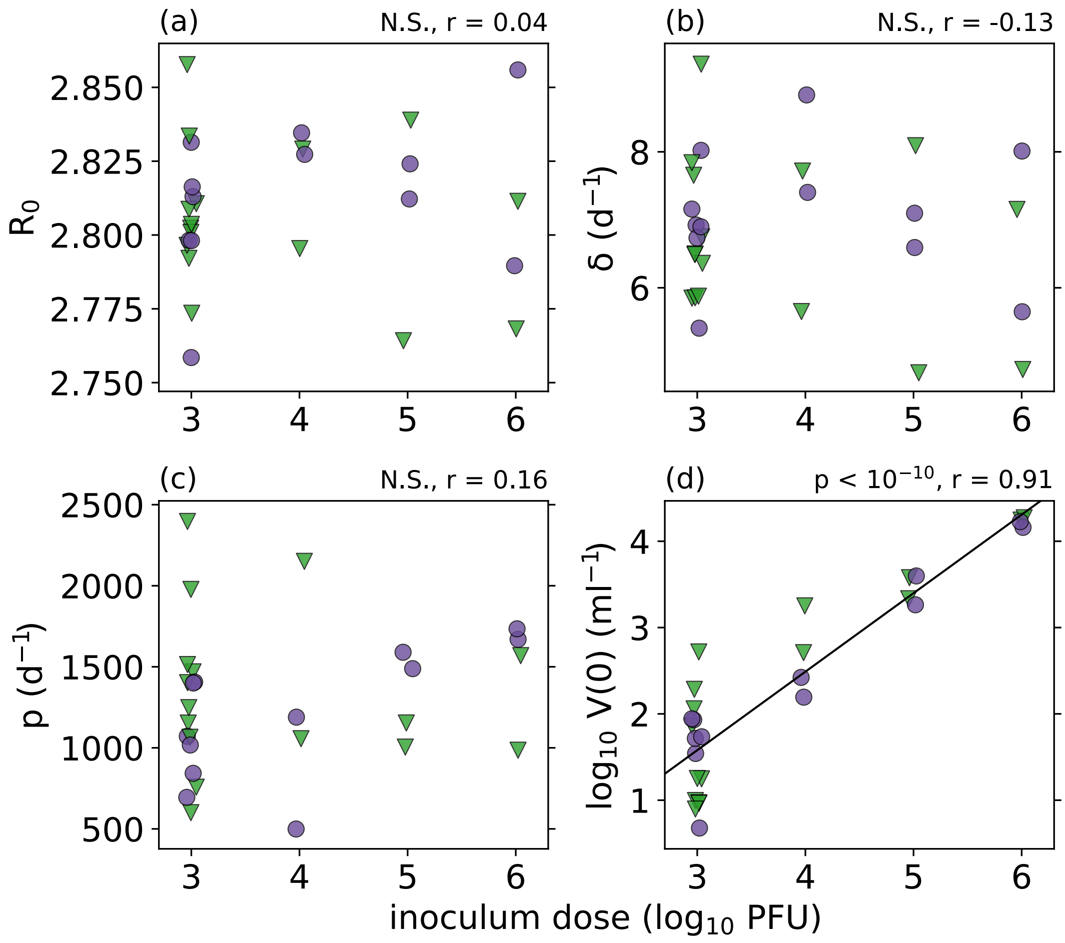

Supplementary Figure 3

Relationships between individual estimated parameters and inoculum dose. Individual estimated parameters are derived from the population fit of the target cell limited model (Eq. 1) with fixed $k = 8$ d^{-1} and fixed $c = 10$ d^{-1}, without any explicit inclusion of covariate structures between inoculum and parameter value. Correlations between parameters and inoculum dose are assessed via the Pearson correlation, with *p*-value (N.S. indicates non-significant relationship) and correlation coefficient shown above each panel. Where the correlation is statistically significant after Bonferroni correction the linear regression line is shown. Markers for individual animals are colored by the viral strain (BR: green triangles, PR: purple circles).
