# Supplementary material for: Zika virus dynamics: Effects of inoculum dose, the innate immune response and viral interference
Source: PLoS Comput Biol. 2021 Jan 20;17(1):e1008564. doi: 10.1371/journal.pcbi.1008564 (PMC7817008; doi:10.1371/journal.pcbi.1008564)
Supplement: S4 Fig — Individual estimated parameters are derived from the population fit of the target cell limited model (Eq 1) with fixed k = 8 d-1 and fixed c = 10 d-1. Differences between parameters by viral strain are assessed by the Mann Whitney U test, with p-value (N.S. indicates non-significant relationship) shown in each panel. Markers for individual animals show the inoculum dose (103 PFU: light blue triangles, 104 PFU: dark blue squares, 105 PFU: orange pentagons, 106 PFU: red hexagons) and the horizontal black line represents the median parameter value for each group. (PDF) [file pcbi.1008564.s012.pdf]

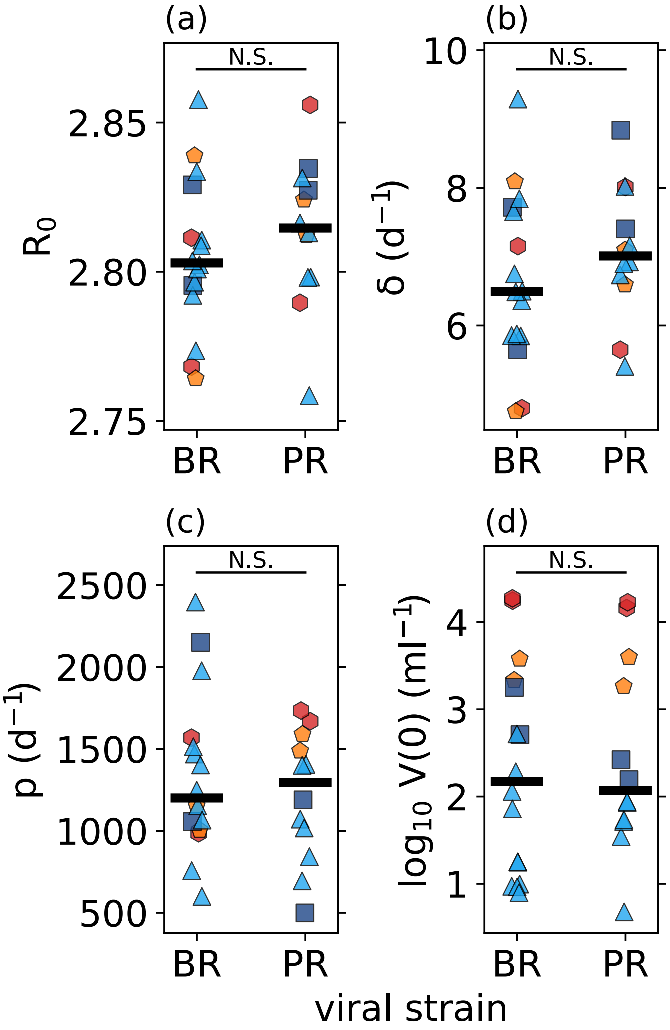

Supplementary Figure 4

Relationships between individual estimated parameters and viral strain. Individual estimated parameters are derived from the population fit of the target cell limited model (Eq. 1) with fixed k = 8 d^{-1} and fixed c = 10 d^{-1}. Differences between parameters by viral strain are assessed by the Mann Whitney U test, with p-value (N.S. indicates non-significant relationship) shown in each panel. Markers for individual animals show the inoculum dose (10^3 PFU: light blue triangles, 10^4 PFU: dark blue squares, 10^5 PFU: orange pentagons, 10^6 PFU: red hexagons) and the horizontal black line represents the median parameter value for each group.
