# Supplementary material for: Zika virus dynamics: Effects of inoculum dose, the innate immune response and viral interference
Source: PLoS Comput Biol. 2021 Jan 20;17(1):e1008564. doi: 10.1371/journal.pcbi.1008564 (PMC7817008; doi:10.1371/journal.pcbi.1008564)
Supplement: S5 Fig — 100,000 repeated random parameter sets were selected from the estimated parameter distributions for each inoculum dose, and predicted viral loads given these parameter values were recorded. Black lines show median predicted viral load, grey shaded region shows 2.5th– 97.5th percentiles of predicted viral loads and black points indicate experimentally observed viral loads. The limit of detection of the experimental assay is shown with a horizontal dashed line and where experimental measurements failed to detect ZIKV in a sample it is shown with an open marker at this limit of detection. (PDF) [file pcbi.1008564.s013.pdf]

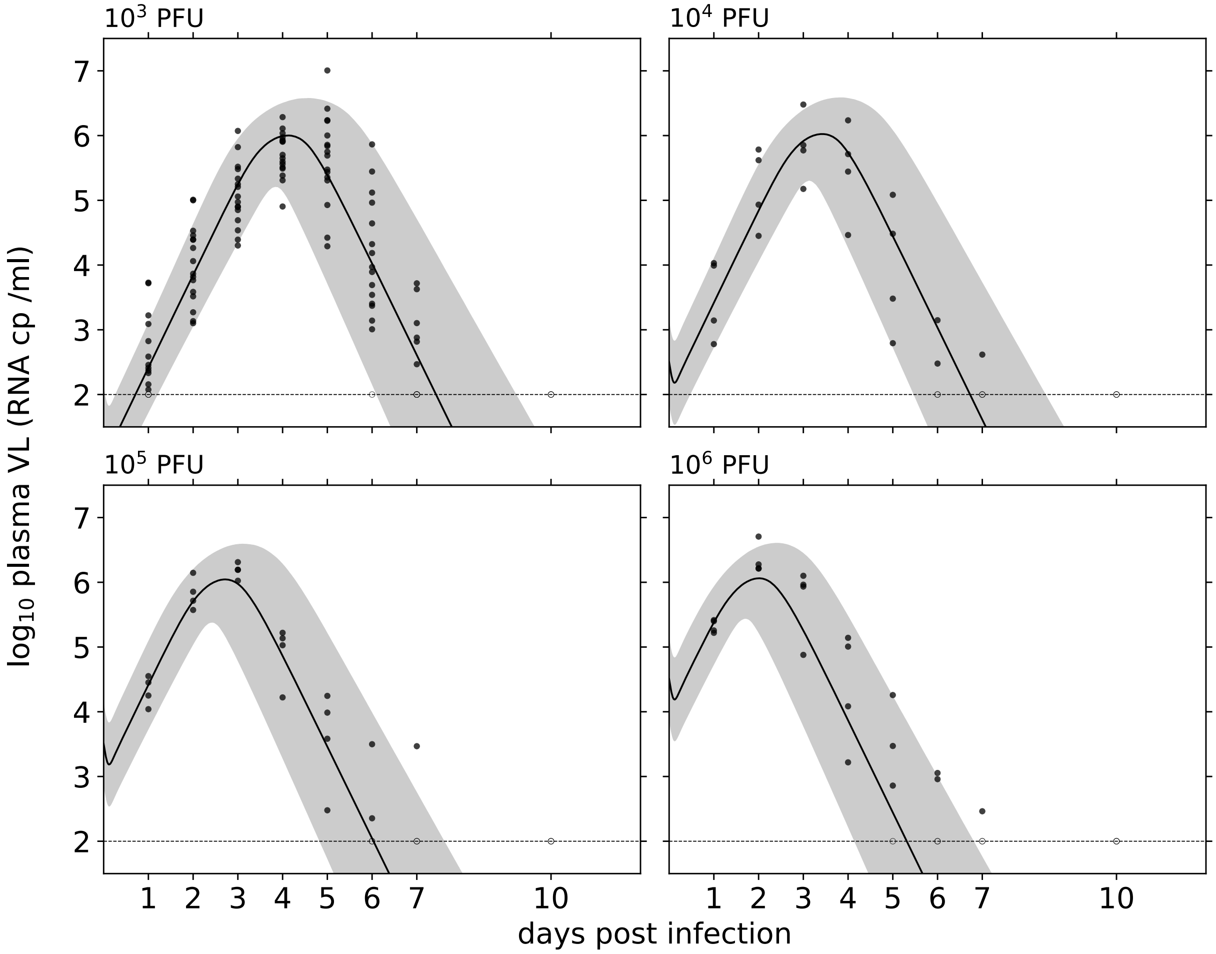

Supplementary Figure 5

Visual predictive checks for the fit of the target cell limited model (Eq. 1) with fixed $k = 8$ d^{-1} and fixed $c = 10$ d^{-1} and with a dose-dependency in $\log_{10} V_0$ explicitly incorporated (Table 1). 100,000 repeated random parameter sets were selected from the estimated parameter distributions for each inoculum dose, and predicted viral loads given these parameter values were recorded. Black lines show median predicted viral load, grey shaded region shows $2.5^{th} - 97.5^{th}$ percentiles of predicted viral loads and black points indicate experimentally observed viral loads. The limit of detection of the experimental assay is shown with a horizontal dashed line and where experimental measurements failed to detect ZIKV in a sample it is shown with an open marker at this limit of detection.
