# Supplementary material for: Zika virus dynamics: Effects of inoculum dose, the innate immune response and viral interference
Source: PLoS Comput Biol. 2021 Jan 20;17(1):e1008564. doi: 10.1371/journal.pcbi.1008564 (PMC7817008; doi:10.1371/journal.pcbi.1008564)
Supplement: S6 Fig — Inoculum dose indicated by color where relevant (light blue = 103 PFU, dark blue = 104 PFU, orange = 105 PFU, red = 106 PFU). (PDF) [file pcbi.1008564.s014.pdf]

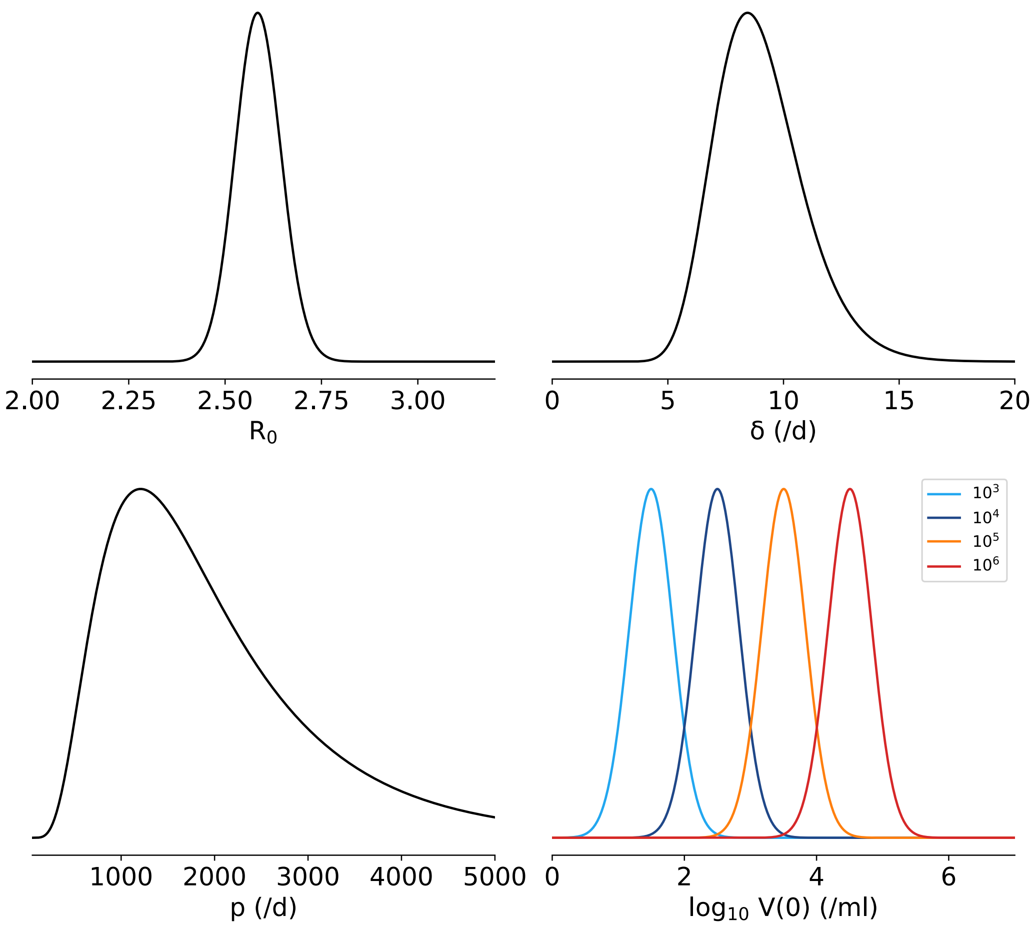


**Supplementary Figure 6**

Estimated parameter distributions from the target cell limited model (Eq. 1) with fixed $k$ = 8 $d^{-1}$ and fixed $c$ = 10 $d^{-1}$ and with a dose-dependency in $\log_{10} V_0$ explicitly incorporated (Table 1). Inoculum dose indicated by color where relevant (light blue = $10^3$ PFU, dark blue = $10^4$ PFU, orange = $10^5$ PFU, red = $10^6$ PFU).
