# Supplementary material for: Zika virus dynamics: Effects of inoculum dose, the innate immune response and viral interference
Source: PLoS Comput Biol. 2021 Jan 20;17(1):e1008564. doi: 10.1371/journal.pcbi.1008564 (PMC7817008; doi:10.1371/journal.pcbi.1008564)
Supplement: S9 Fig — Color and marker shape indicate inoculum strain (BR: green triangles, PR: purple circles) and inoculum dose is indicated top left of each panel. Observed VLs are shown by markers and model prediction is shown by the solid line. The limit of detection of the experimental assay is shown by the horizontal dashed line and where ZIKV is not detectable in a sample it is shown with an open marker at this value. (PDF) [file pcbi.1008564.s017.pdf]

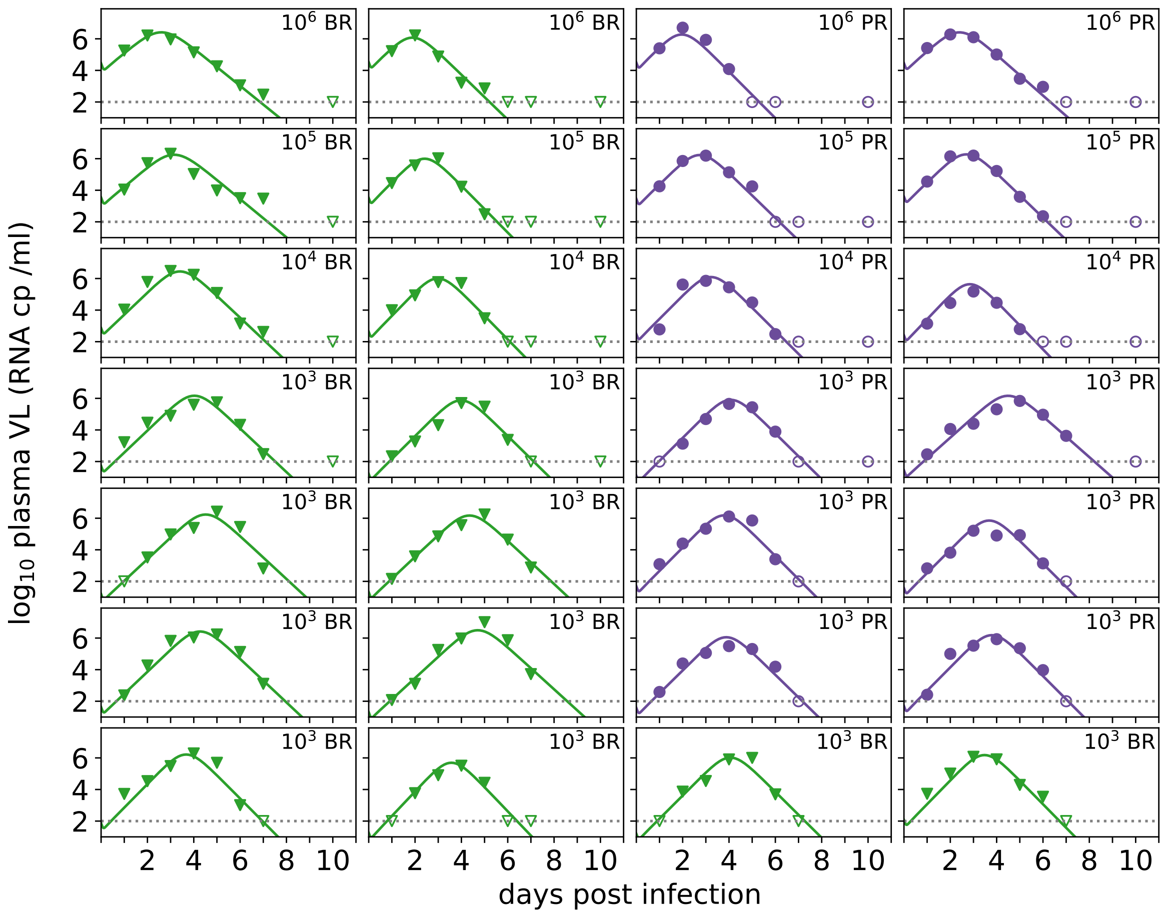

Supplementary Figure 9

Predicted viral loads for each individual animal from the fit of the target cell limited model (Eq. 1) with fixed k = 8 d^{-1} and fixed c = 10 d^{-1} and with a dose-dependency in $\log_{10}$ V_0 explicitly incorporated (Table 1). Color and marker shape indicate inoculum strain (BR: green triangles, PR: purple circles) and inoculum dose is indicated top left of each panel. Observed VLs are shown by markers and model prediction is shown by the solid line. The limit of detection of the experimental assay is shown by the horizontal dashed line and where ZIKV is not detectable in a sample it is shown with an open marker at this value.
