# Supplementary material for: Zika virus dynamics: Effects of inoculum dose, the innate immune response and viral interference
Source: PLoS Comput Biol. 2021 Jan 20;17(1):e1008564. doi: 10.1371/journal.pcbi.1008564 (PMC7817008; doi:10.1371/journal.pcbi.1008564)
Supplement: S10 Fig — Model fits are of the target cell limited model (Eq 1) with fixed k = 8 d-1 and fixed c = 10 d-1 and with a dose-dependency in log10 V0 explicitly incorporated. Each circle represents the output from a repeated fit with randomly selected initial parameter guesses and random seeds. The horizontal line shows the log-likelihood from the model fit with lognormally distributed δ. (PDF) [file pcbi.1008564.s018.pdf]

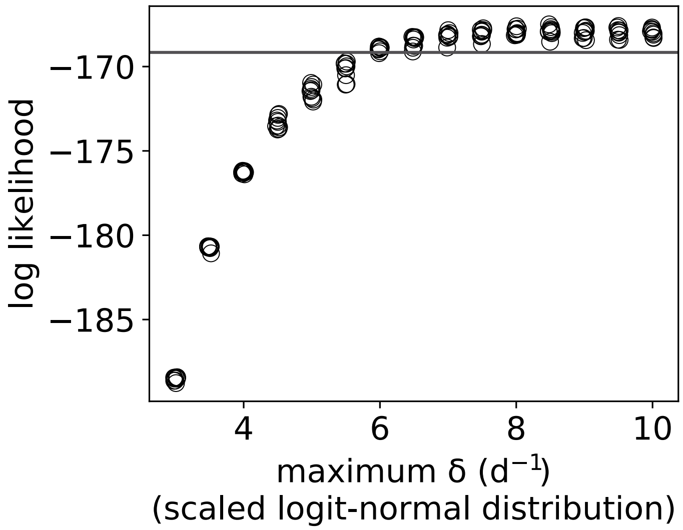

Supplementary Figure 10

The log likelihood from model fits where the population distribution of δ is a scaled logit-normal, so it is only able to take values up to a maximum, rather than a lognormal as for other parameters. Model fits are of the target cell limited model (Eq. 1) with fixed k = 8 d^{-1} and fixed c = 10 d^{-1} and with a dose-dependency in $\log_{10} V_0$ explicitly incorporated. Each circle represents the output from a repeated fit with randomly selected initial parameter guesses and random seeds. The horizontal line shows the log-likelihood from the model fit with lognormally distributed δ.
