# Supplementary material for: Zika virus dynamics: Effects of inoculum dose, the innate immune response and viral interference
Source: PLoS Comput Biol. 2021 Jan 20;17(1):e1008564. doi: 10.1371/journal.pcbi.1008564 (PMC7817008; doi:10.1371/journal.pcbi.1008564)
Supplement: S12 Fig — Inoculum dose indicated by color where relevant (light blue = 103 PFU, dark blue = 104 PFU, orange = 105 PFU, red = 106 PFU). (PDF) [file pcbi.1008564.s020.pdf]

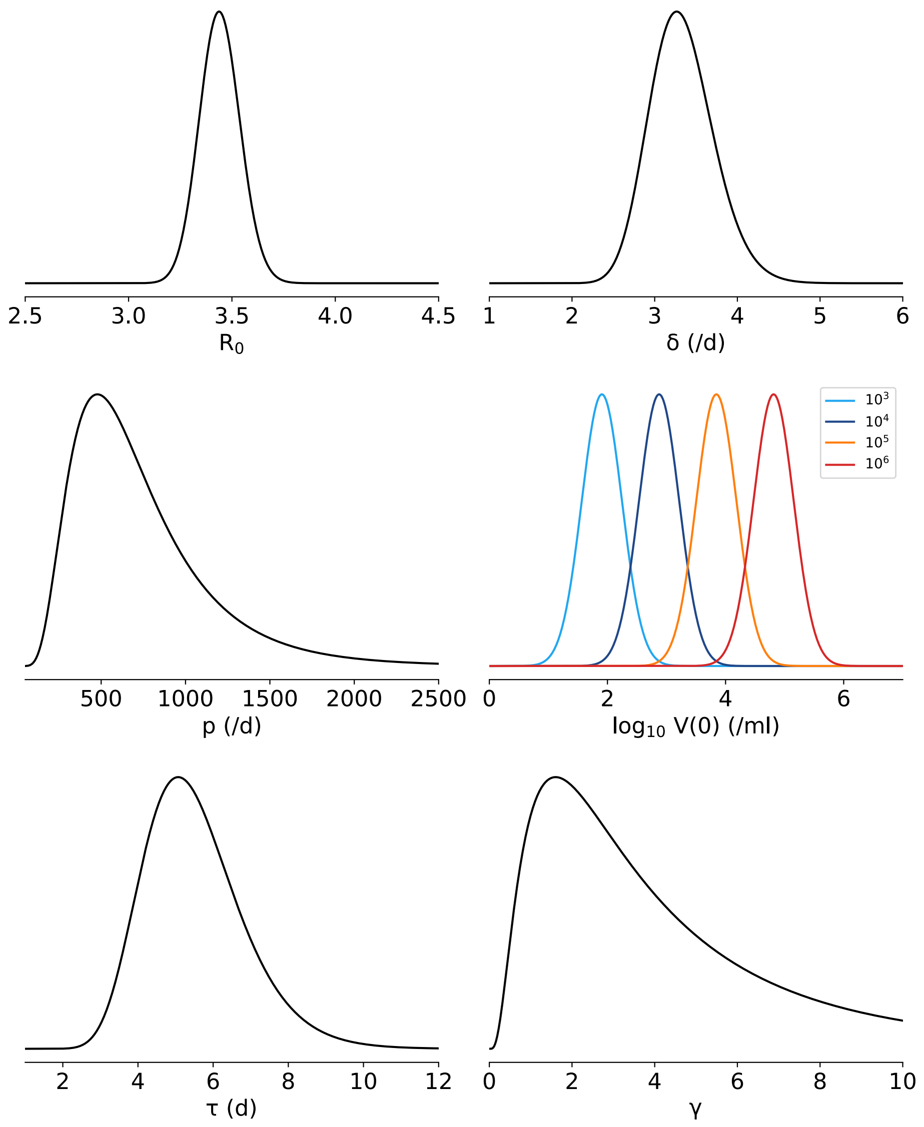

Supplementary Figure 12

Estimated parameter distributions from the innate immune model with reduced viral production rate (Eq. 2) with fixed $k = 8$ d^{-1}, fixed $c = 10$ d^{-1}, fixed $s = 1$ d^{-1} and fixed $\alpha = 2$ d^{-1}, and with a dose-dependency in $\log_{10} V_0$ explicitly incorporated (Supplementary Table 4). Inoculum dose indicated by color where relevant (light blue = 10^3 PFU, dark blue = 10^4 PFU, orange = 10^5 PFU, red = 10^6 PFU).
