# Supplementary material for: Zika virus dynamics: Effects of inoculum dose, the innate immune response and viral interference
Source: PLoS Comput Biol. 2021 Jan 20;17(1):e1008564. doi: 10.1371/journal.pcbi.1008564 (PMC7817008; doi:10.1371/journal.pcbi.1008564)
Supplement: S14 Fig — Differences between parameters by viral strain are assessed by the Mann Whitney U test, and no significant relationships after Bonferroni correction are observed. Markers for individual animals are colored by the viral strain (BR: green triangles, PR: purple circles). (PDF) [file pcbi.1008564.s022.pdf]

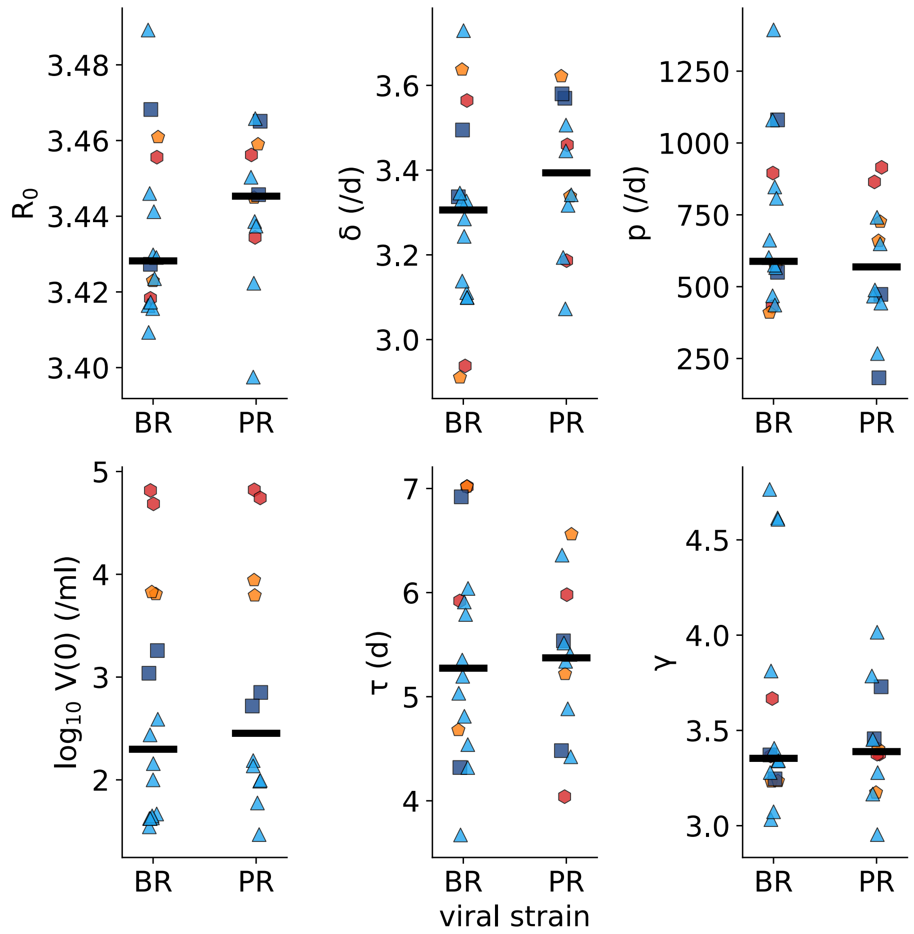

Supplementary Figure 14

Relationships between individual estimated parameters and viral strain, with individual estimated parameters from the innate immune model with reduced viral production rate (Eq. 2) with fixed $k = 8$ d^{-1}, fixed $c = 10$ d^{-1}, fixed $s = 1$ d^{-1} and fixed $\alpha = 2$ d^{-1}, and with a dose-dependency in $\log_{10} V_0$ explicitly incorporated (Supplementary Table 4). Differences between parameters by viral strain are assessed by the Mann Whitney U test, and no significant relationships after Bonferroni correction are observed. Markers for individual animals are colored by the viral strain (BR: green triangles, PR: purple circles).
