# Supplementary material for: Zika virus dynamics: Effects of inoculum dose, the innate immune response and viral interference
Source: PLoS Comput Biol. 2021 Jan 20;17(1):e1008564. doi: 10.1371/journal.pcbi.1008564 (PMC7817008; doi:10.1371/journal.pcbi.1008564)
Supplement: S15 Fig — Color and marker shape indicate inoculum strain (BR: green triangles, PR: purple circles) and inoculum dose is indicated top left of each panel. Observed VLs are shown by markers and model prediction is shown by the solid line. The limit of detection of the experimental assay is shown by the horizontal dashed line and where ZIKV is not detectable in a sample it is shown with an open marker at this value. (PDF) [file pcbi.1008564.s023.pdf]

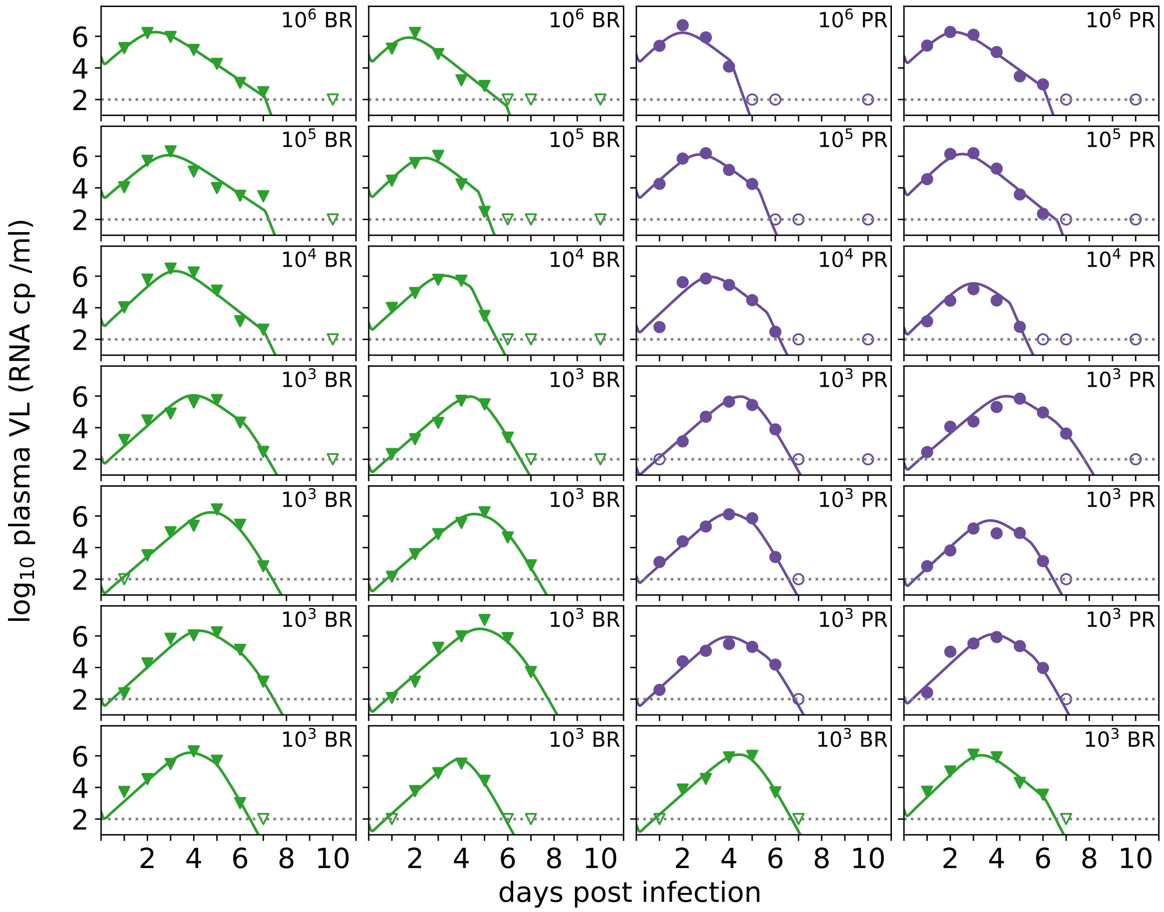

Supplementary Figure 15

Predicted viral loads for each individual animal from the fit of the innate immune model with reduced viral production rate (Eq. 2) with fixed $k = 8$ d^{-1}, fixed $c = 10$ d^{-1}, fixed $s = 1$ d^{-1} and fixed $\alpha = 2$ d^{-1}, and with a dose-dependency in $\log_{10} V_0$ explicitly incorporated (Supplementary Table 4). Color and marker shape indicate inoculum strain (BR: green triangles, PR: purple circles) and inoculum dose is indicated top left of each panel. Observed VLs are shown by markers and model prediction is shown by the solid line. The limit of detection of the experimental assay is shown by the horizontal dashed line and where ZIKV is not detectable in a sample it is shown with an open marker at this value.
