# Supplementary material for: Zika virus dynamics: Effects of inoculum dose, the innate immune response and viral interference
Source: PLoS Comput Biol. 2021 Jan 20;17(1):e1008564. doi: 10.1371/journal.pcbi.1008564 (PMC7817008; doi:10.1371/journal.pcbi.1008564)
Supplement: S16 Fig — Each circle represents the model fit from one implementation of the fitting algorithm with randomly selected initial guesses and random seed. In the likelihood panel, the horizontal line shows the maximum likelihood with fixed α = 2 d-1 (S9 Fig and Table 1). The p-values (N.S denotes non-significance after Bonferroni correction) above each panel are from a Friedman test for repeated measurements. (PDF) [file pcbi.1008564.s024.pdf]

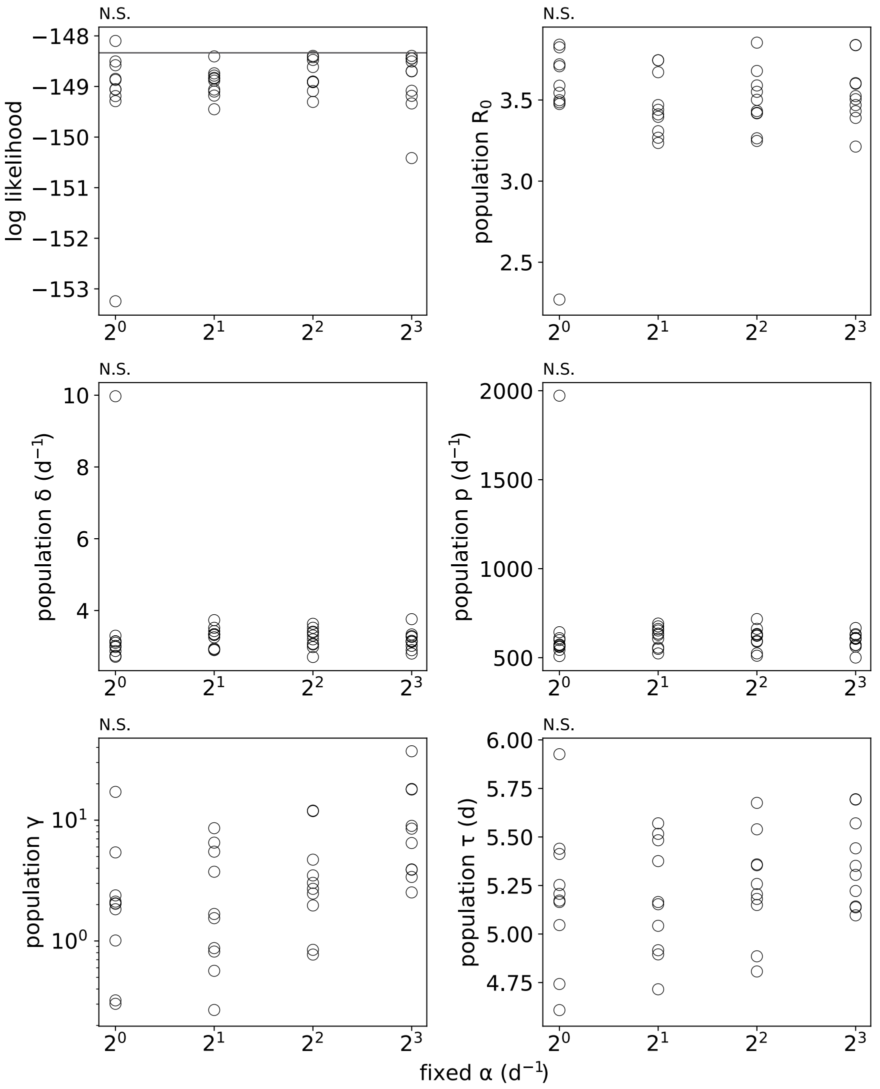

Supplementary Figure 16

Estimated log likelihood and population parameters (Table 1) of the innate immune model with reduced viral production rate (Eq. 2) with fixed $k = 8$ d^{-1}, fixed $c = 10$ d^{-1}, fixed $s = 1$ d^{-1} and fixed α as indicated on the horizontal axis. Each circle represents the model fit from one implementation of the fitting algorithm with randomly selected initial guesses and random seed. In the likelihood panel, the horizontal line shows the maximum likelihood with fixed $\alpha = 2$ d^{-1} (Supplementary Figure 9 and Table 1). The p-values (N.S denotes non-significance after Bonferroni correction) above each panel are from a Friedman test for repeated measurements.
