# Supplementary material for: Zika virus dynamics: Effects of inoculum dose, the innate immune response and viral interference
Source: PLoS Comput Biol. 2021 Jan 20;17(1):e1008564. doi: 10.1371/journal.pcbi.1008564 (PMC7817008; doi:10.1371/journal.pcbi.1008564)
Supplement: S22 Fig — (PDF) [file pcbi.1008564.s030.pdf]

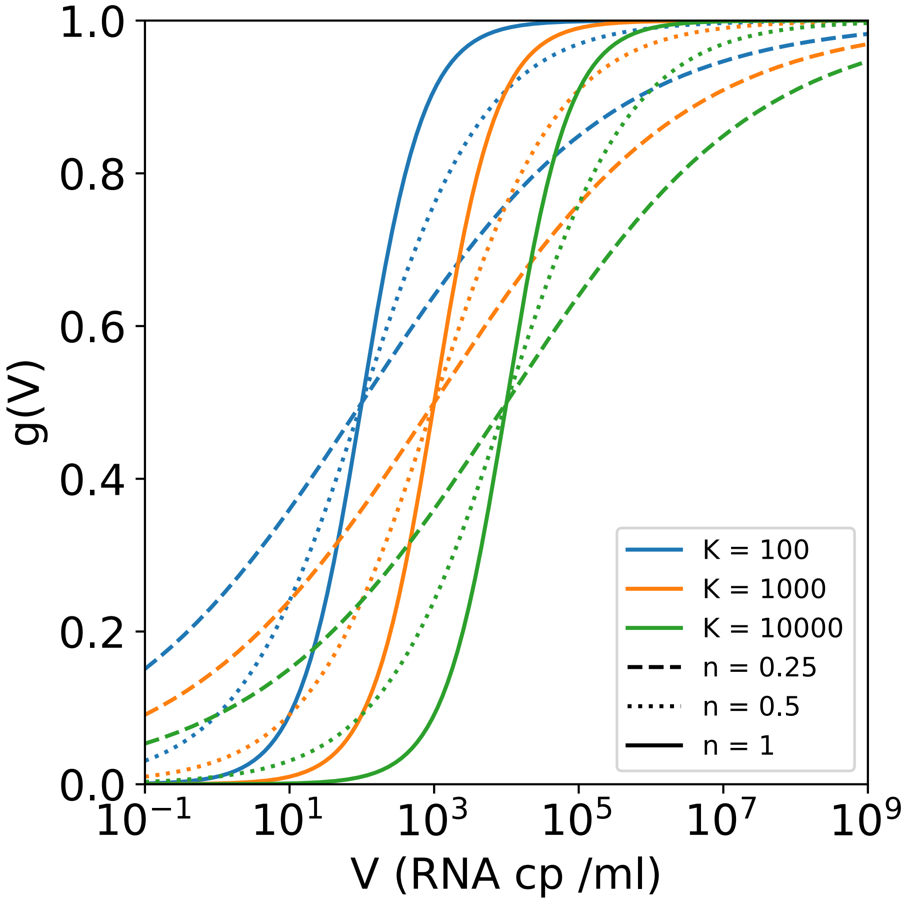


**Supplementary Figure 22**

The relationship between viral load $V$ (x-axis) and viral interference $g(V)$ in the viral interference model (Eq. 3).
