# Supplementary material for: Zika virus dynamics: Effects of inoculum dose, the innate immune response and viral interference
Source: PLoS Comput Biol. 2021 Jan 20;17(1):e1008564. doi: 10.1371/journal.pcbi.1008564 (PMC7817008; doi:10.1371/journal.pcbi.1008564)
Supplement: S23 Fig — Those fits which are indistinguishable by log likelihood, within 2 points of the maximum, are highlighted in white. (PDF) [file pcbi.1008564.s031.pdf]

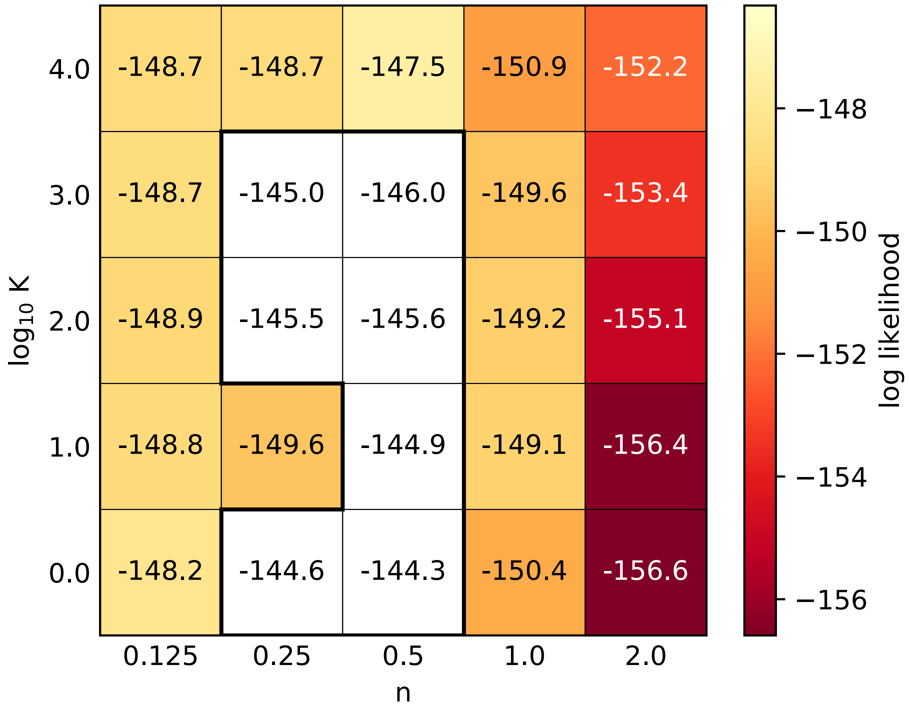

Supplementary Figure 23

Maximum log likelihoods, coloured by value, found from fitting the viral interference model (Eq. 3) to observed viral load data with different values of K (the half maximal response parameter) and n (the Hill coefficient). Those fits which are indistinguishable by log likelihood, within 2 points of the maximum, are highlighted in white.
