# Supplementary material for: Zika virus dynamics: Effects of inoculum dose, the innate immune response and viral interference
Source: PLoS Comput Biol. 2021 Jan 20;17(1):e1008564. doi: 10.1371/journal.pcbi.1008564 (PMC7817008; doi:10.1371/journal.pcbi.1008564)
Supplement: S26 Fig — Correlations between parameters and inoculum dose are assessed via the Pearson correlation, and where this is significant after Bonferroni correction the linear regression line is shown (dashed) and the p-value is shown above the panel. Markers for individual animals are colored by the viral strain (BR: green triangles, PR: purple circles). (PDF) [file pcbi.1008564.s034.pdf]

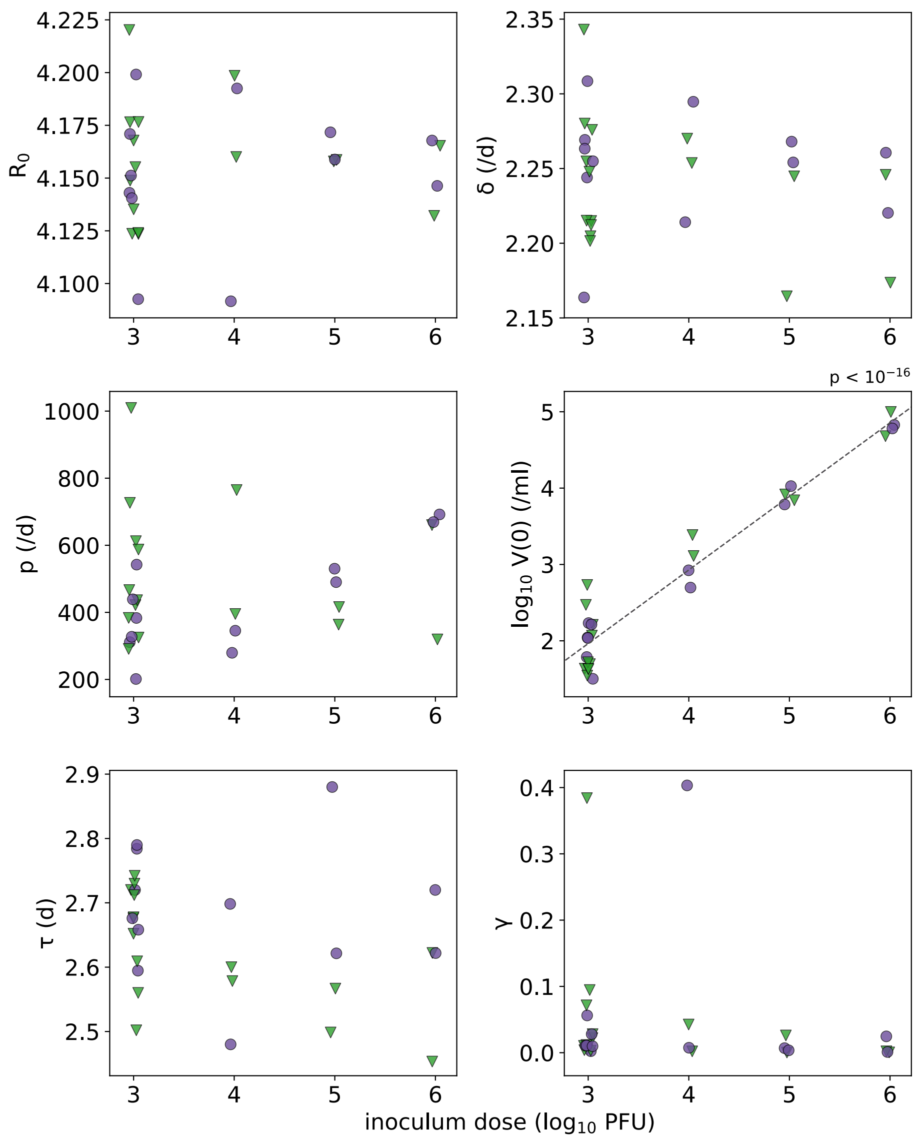

Supplementary Figure 26

Relationships between individual estimated parameters and inoculum dose, with individual estimated parameters from the viral interference model (Eq. 3) with fixed $k = 8$ d^{-1}, fixed $c = 10$ d^{-1}, fixed $s = 1$ d^{-1}, fixed $\alpha = 2$ d^{-1}, fixed $K = 10^3$ and fixed $n = 0.25$, and with a dose-dependency in $\log_{10} V_0$ explicitly incorporated (Table 1). Correlations between parameters and inoculum dose are assessed via the Pearson correlation, and where this is significant after Bonferroni correction the linear regression line is shown (dashed) and the p-value is shown above the panel. Markers for individual animals are colored by the viral strain (BR: green triangles, PR: purple circles).
