# Supplementary material for: Zika virus dynamics: Effects of inoculum dose, the innate immune response and viral interference
Source: PLoS Comput Biol. 2021 Jan 20;17(1):e1008564. doi: 10.1371/journal.pcbi.1008564 (PMC7817008; doi:10.1371/journal.pcbi.1008564)
Supplement: S27 Fig — Differences between parameters by viral strain are assessed by the Mann Whitney U test, and no significant relationships after Bonferroni correction are observed. Markers for individual animals are colored by the viral strain (BR: green triangles, PR: purple circles). (PDF) [file pcbi.1008564.s035.pdf]

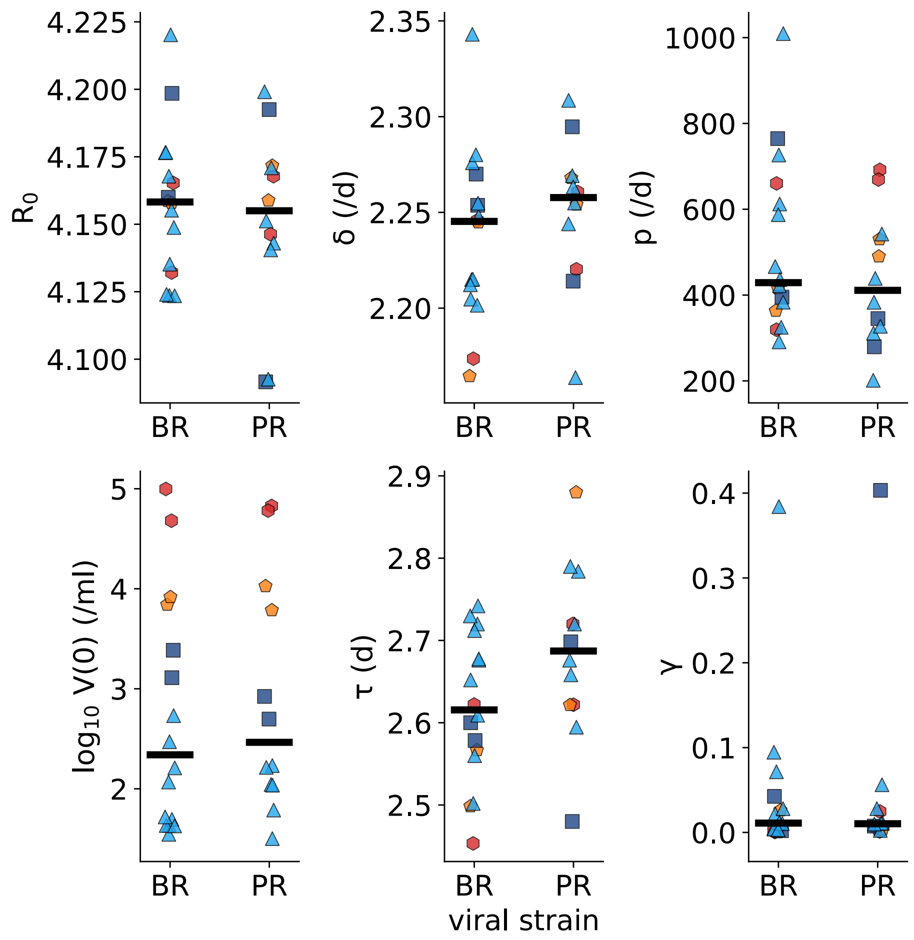

Supplementary Figure 27

Relationships between individual estimated parameters and viral strain, with individual estimated parameters from the viral interference model (Eq. 3) with fixed $k = 8$ d^{-1}, fixed $c = 10$ d^{-1}, fixed $s = 1$ d^{-1}, fixed $\alpha = 2$ d^{-1}, fixed $K = 10^3$ and fixed $n = 0.25$, and with a dose-dependency in $\log_{10} V_0$ explicitly incorporated (Table 1). Differences between parameters by viral strain are assessed by the Mann Whitney U test, and no significant relationships after Bonferroni correction are observed. Markers for individual animals are colored by the viral strain (BR: green triangles, PR: purple circles).
