# Supplementary material for: Zika virus dynamics: Effects of inoculum dose, the innate immune response and viral interference
Source: PLoS Comput Biol. 2021 Jan 20;17(1):e1008564. doi: 10.1371/journal.pcbi.1008564 (PMC7817008; doi:10.1371/journal.pcbi.1008564)
Supplement: S29 Fig — Top: the area under the curve (AUC) of the immune-response-restricted viral production rate (p^(t)) normalized by the estimated viral production rate in the absence of immune response (p) for each animal. Middle: The maximum effect of immune response on viral production rate, with 1 representing complete control of viral production, 0 representing no restriction of viral production. Bottom: the AUC of the total viral production, given by the immune restricted viral production rate p^(t) multiplied by the productive infected cell concentration I2(t). In each panel, the p-value shown is from a linear regression and where this is statistically significant (p < 0.05) the linear regression line is shown. (PDF) [file pcbi.1008564.s037.pdf]

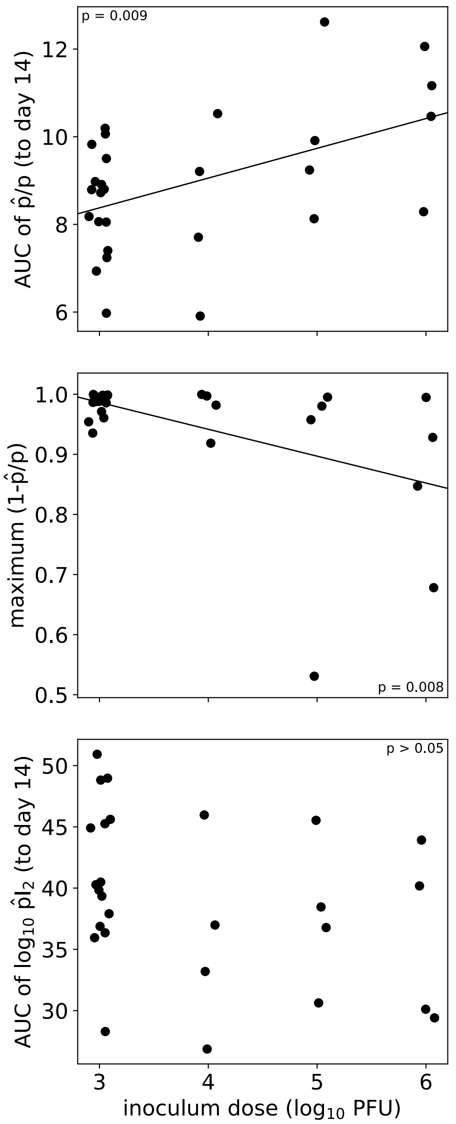

Supplementary Figure 29

Immune response summary quantities by inoculum dose for the viral interference model (Eq 3, Table 1). Top: the area under the curve (AUC) of the immune-response-restricted viral production rate ($\hat{p}(t)$) normalised by the estimated viral production rate in the absence of immune response (p) for each animal. Middle: The maximum effect of immune response on viral production rate, with 1 representing complete control of viral production, 0 representing no restriction of viral production. Bottom: the AUC of the total viral production, given by the immune restricted viral production rate $\hat{p}(t)$ multiplied by the productive infected cell concentration I_2(t).

In each panel, the p-value shown is from a linear regression and where this is statistically significant ($p < 0.05$) the linear regression line is shown.
