# Supplementary material for: Zika virus dynamics: Effects of inoculum dose, the innate immune response and viral interference
Source: PLoS Comput Biol. 2021 Jan 20;17(1):e1008564. doi: 10.1371/journal.pcbi.1008564 (PMC7817008; doi:10.1371/journal.pcbi.1008564)
Supplement: S30 Fig — (PDF) [file pcbi.1008564.s038.pdf]

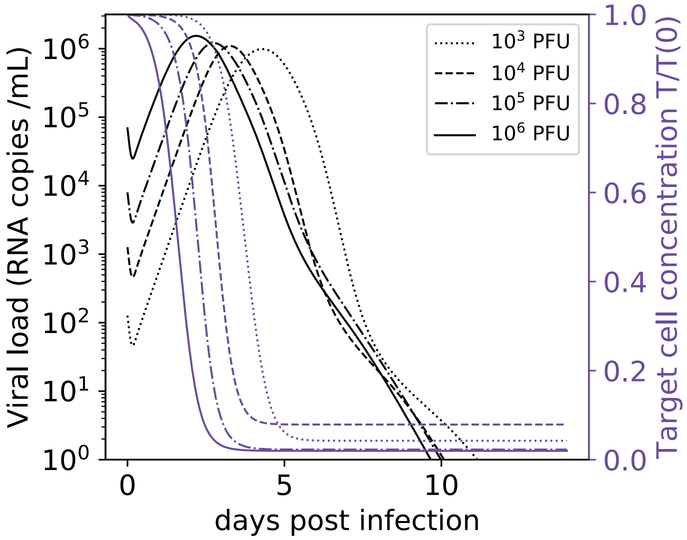

Supplementary Figure 30

The mean predicted viral load (left axis, black) and mean predicted target cell concentration normalised by T(0) (purple, right axis) at each dose group, from the innate immune model with restricted viral production and viral interference (Eq. 3, Table 1).
