# Supplementary material for: Zika virus dynamics: Effects of inoculum dose, the innate immune response and viral interference
Source: PLoS Comput Biol. 2021 Jan 20;17(1):e1008564. doi: 10.1371/journal.pcbi.1008564 (PMC7817008; doi:10.1371/journal.pcbi.1008564)
Supplement: S31 Fig — Correlations between inoculum dose and viral characteristic are assessed via a Pearson correlation, with p-value shown in each panel. Where this relationship is found to be significant at the α = 0.05 level after Bonferroni correction for multiple testing (m = 6) the linear regression line is shown in the panel. The VL AUC and downslopes are calculated as for the observed viral loads, described in Methods. (PDF) [file pcbi.1008564.s039.pdf]

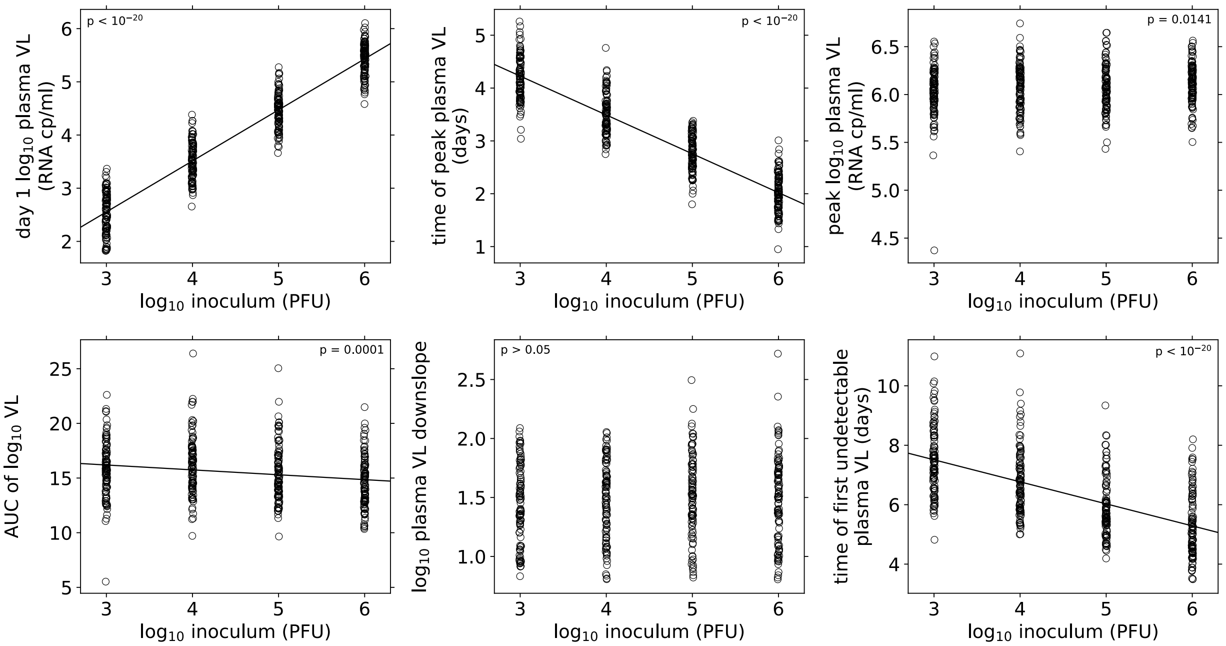

Supplementary Figure 31

Viral load (VL) characteristics from 100 simulated viral dynamics profiles with model parameters selected from the fit of the viral interference model (Eq. 3, Table 1). Correlations between inoculum dose and viral characteristic are assessed via a Pearson correlation, with p-value shown in each panel. Where this relationship is found to be significant at the $\alpha = 0.05$ level after Bonferroni correction for multiple testing ($m = 6$) the linear regression line is shown in the panel. The VL AUC and downslopes are calculated as for the observed viral loads, described in Methods.
